# Supplementary material for: Glucosinolate Profiling and Expression Analysis of Glucosinolate Biosynthesis Genes Differentiate White Mold Resistant and Susceptible Cabbage Lines
Source: Int J Mol Sci. 2018 Dec 13;19(12):4037. doi: 10.3390/ijms19124037 (PMC6321582; doi:10.3390/ijms19124037)
Supplement: Supplementary file 1 [file ijms-19-04037-s001.zip › Supplementary Figures.pptx]

## Slide 1
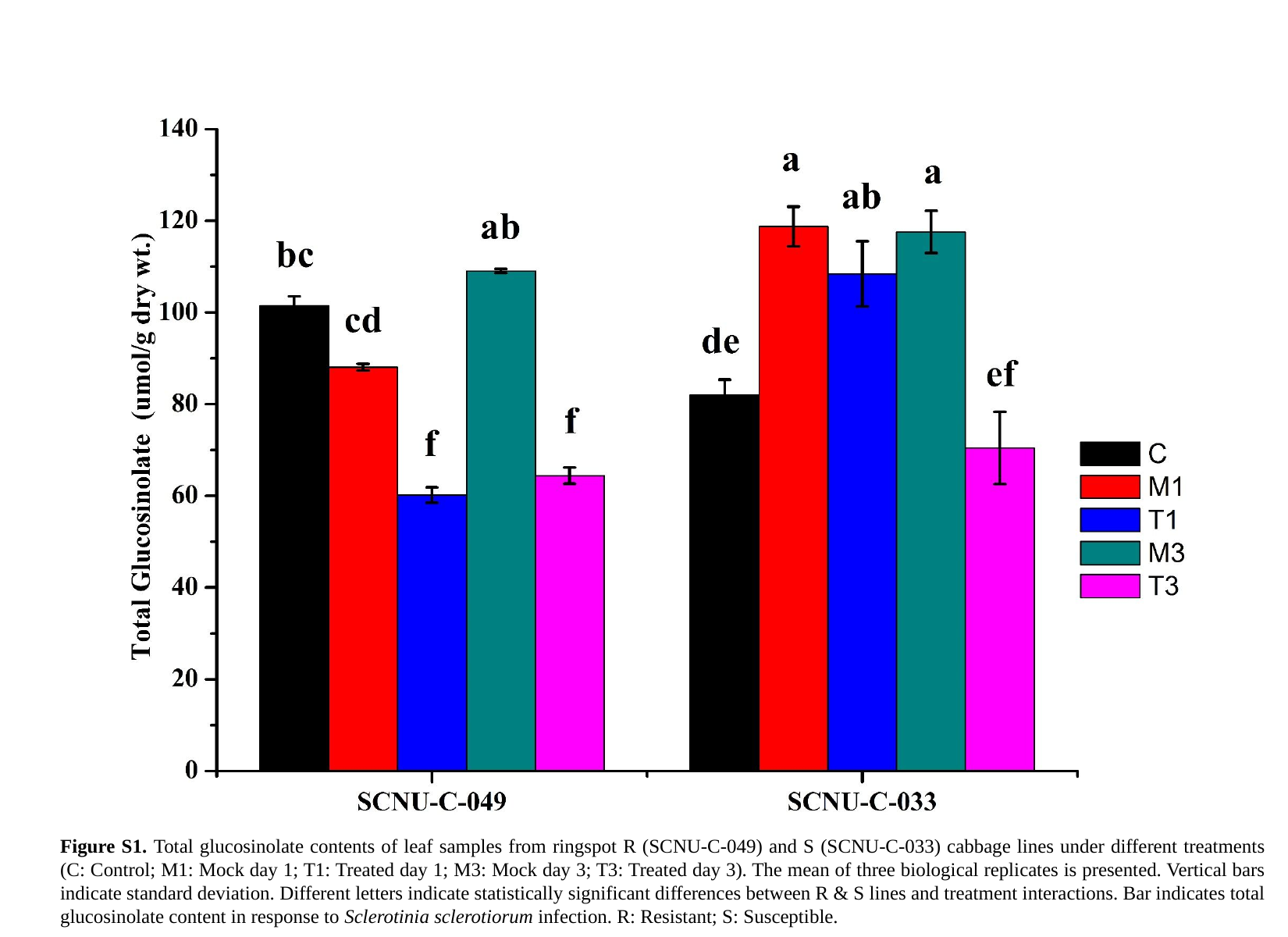

Figure S1. Total glucosinolate contents of leaf samples from ringspot R (SCNU-C-049) and S (SCNU-C-033) cabbage lines under different treatments (C: Control; M1: Mock day 1; T1: Treated day 1; M3: Mock day 3; T3: Treated day 3). The mean of three biological replicates is presented. Vertical bars indicate standard deviation. Different letters indicate statistically significant differences between R & S lines and treatment interactions. Bar indicates total glucosinolate content in response to Sclerotinia sclerotiorum infection. R: Resistant; S: Susceptible.

## Slide 2
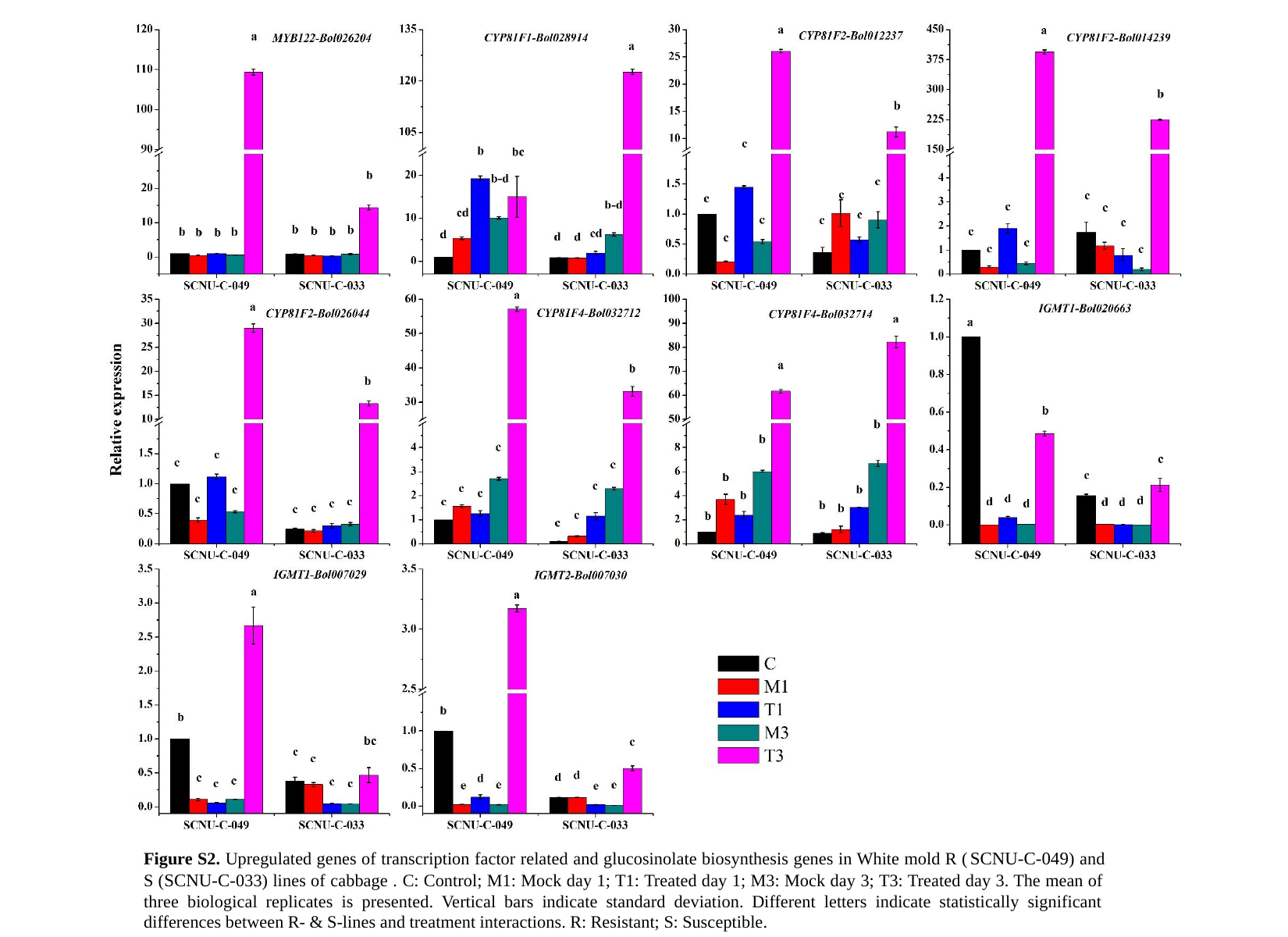

Figure S2. Upregulated genes of transcription factor related and glucosinolate biosynthesis genes in White mold R (SCNU-C-049) and S (SCNU-C-033) lines of cabbage . C: Control; M1: Mock day 1; T1: Treated day 1; M3: Mock day 3; T3: Treated day 3. The mean of three biological replicates is presented. Vertical bars indicate standard deviation. Different letters indicate statistically significant differences between R- & S-lines and treatment interactions. R: Resistant; S: Susceptible.

## Slide 3
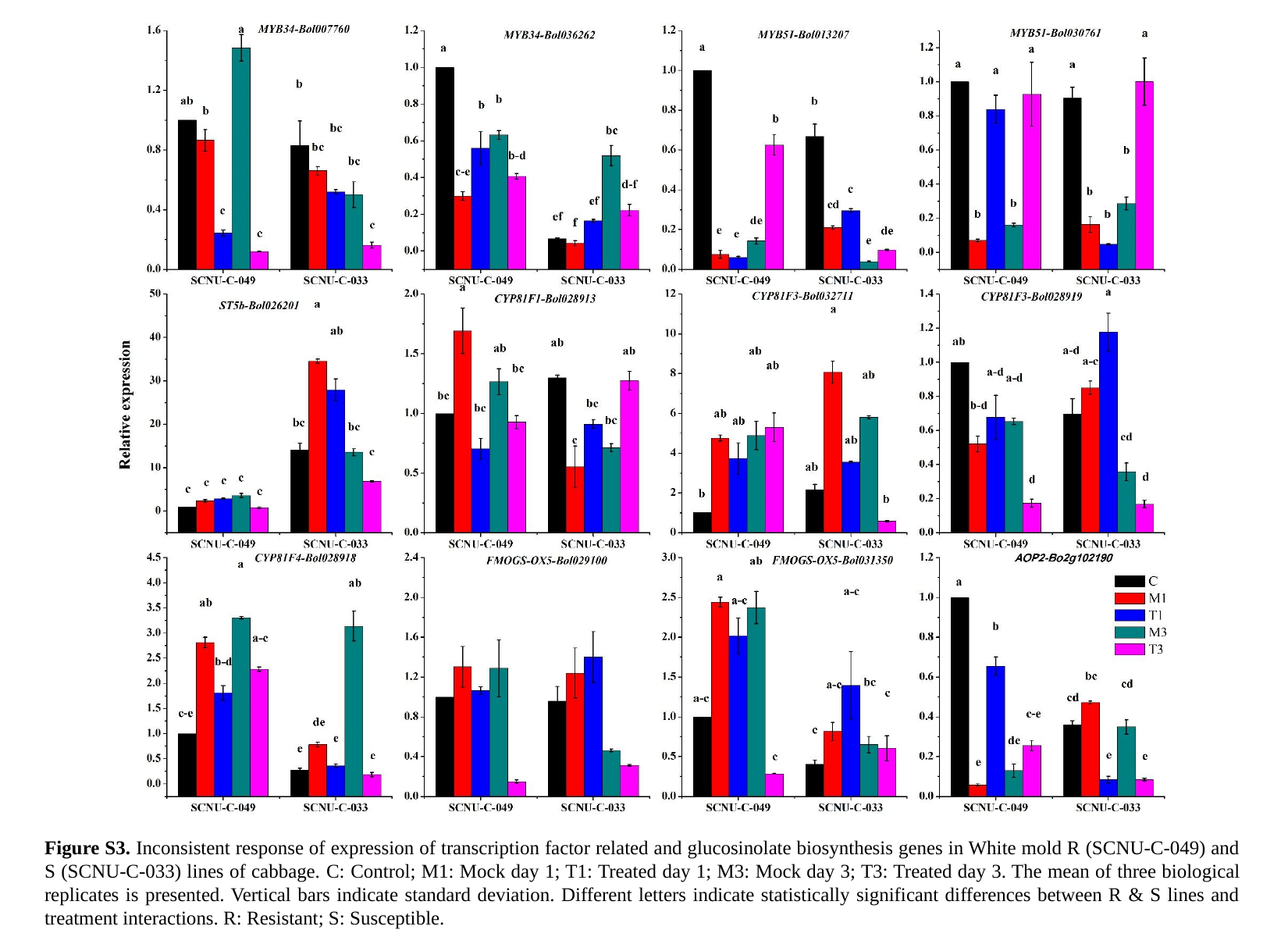

Figure S3. Inconsistent response of expression of transcription factor related and glucosinolate biosynthesis genes in White mold R (SCNU-C-049) and S (SCNU-C-033) lines of cabbage. C: Control; M1: Mock day 1; T1: Treated day 1; M3: Mock day 3; T3: Treated day 3. The mean of three biological replicates is presented. Vertical bars indicate standard deviation. Different letters indicate statistically significant differences between R & S lines and treatment interactions. R: Resistant; S: Susceptible.

## Slide 4
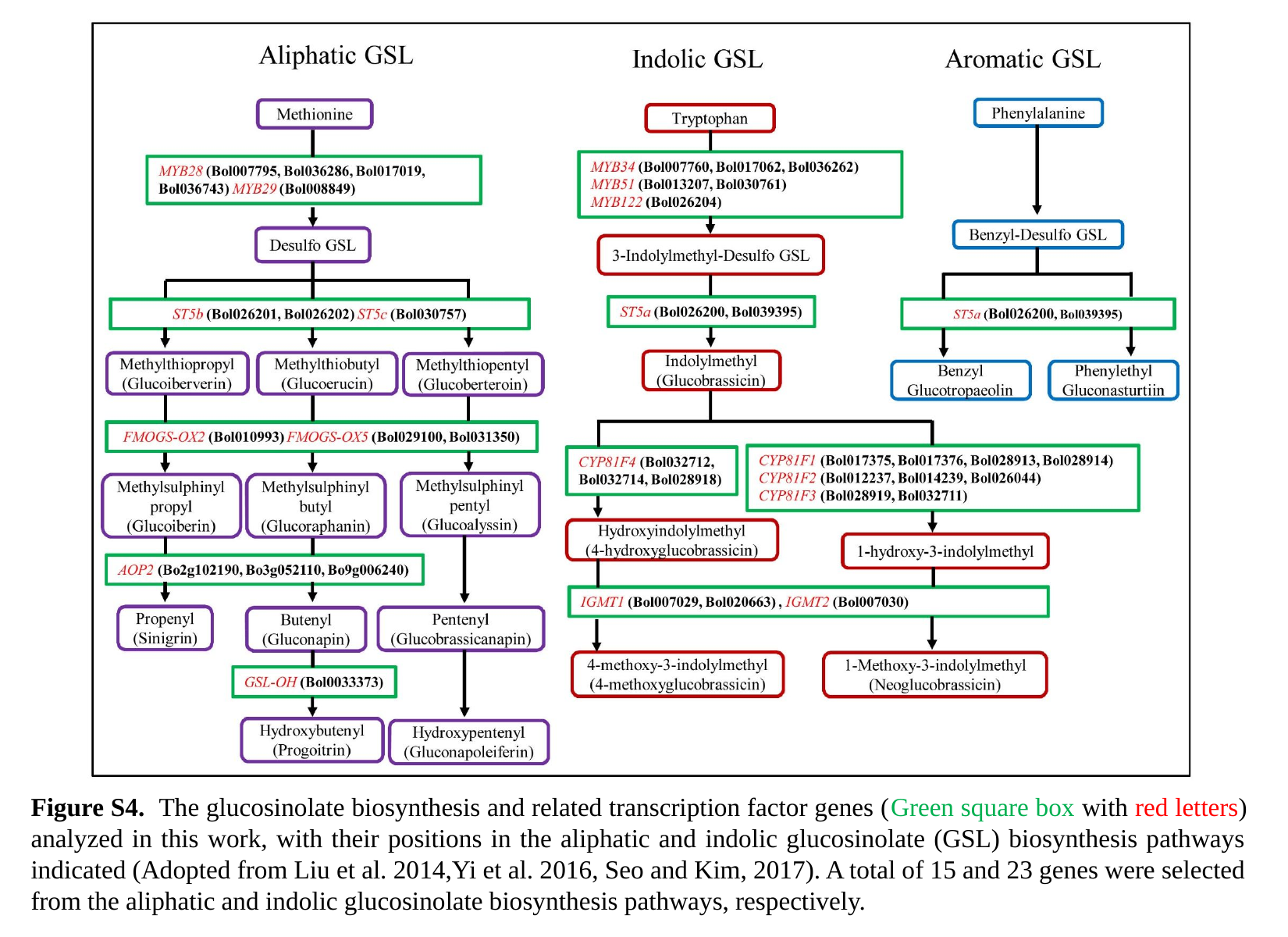

Figure S4. The glucosinolate biosynthesis and related transcription factor genes (Green square box with red letters) analyzed in this work, with their positions in the aliphatic and indolic glucosinolate (GSL) biosynthesis pathways indicated (Adopted from Liu et al. 2014,Yi et al. 2016, Seo and Kim, 2017). A total of 15 and 23 genes were selected from the aliphatic and indolic glucosinolate biosynthesis pathways, respectively.
