# Supplementary material for: Glucosinolate Profiling and Expression Analysis of Glucosinolate Biosynthesis Genes Differentiate White Mold Resistant and Susceptible Cabbage Lines
Source: Int J Mol Sci. 2018 Dec 13;19(12):4037. doi: 10.3390/ijms19124037 (PMC6321582; doi:10.3390/ijms19124037)
Supplement: Supplementary file 1 [file ijms-19-04037-s001.zip › Supplementary file 1.pptx]

## Slide 1
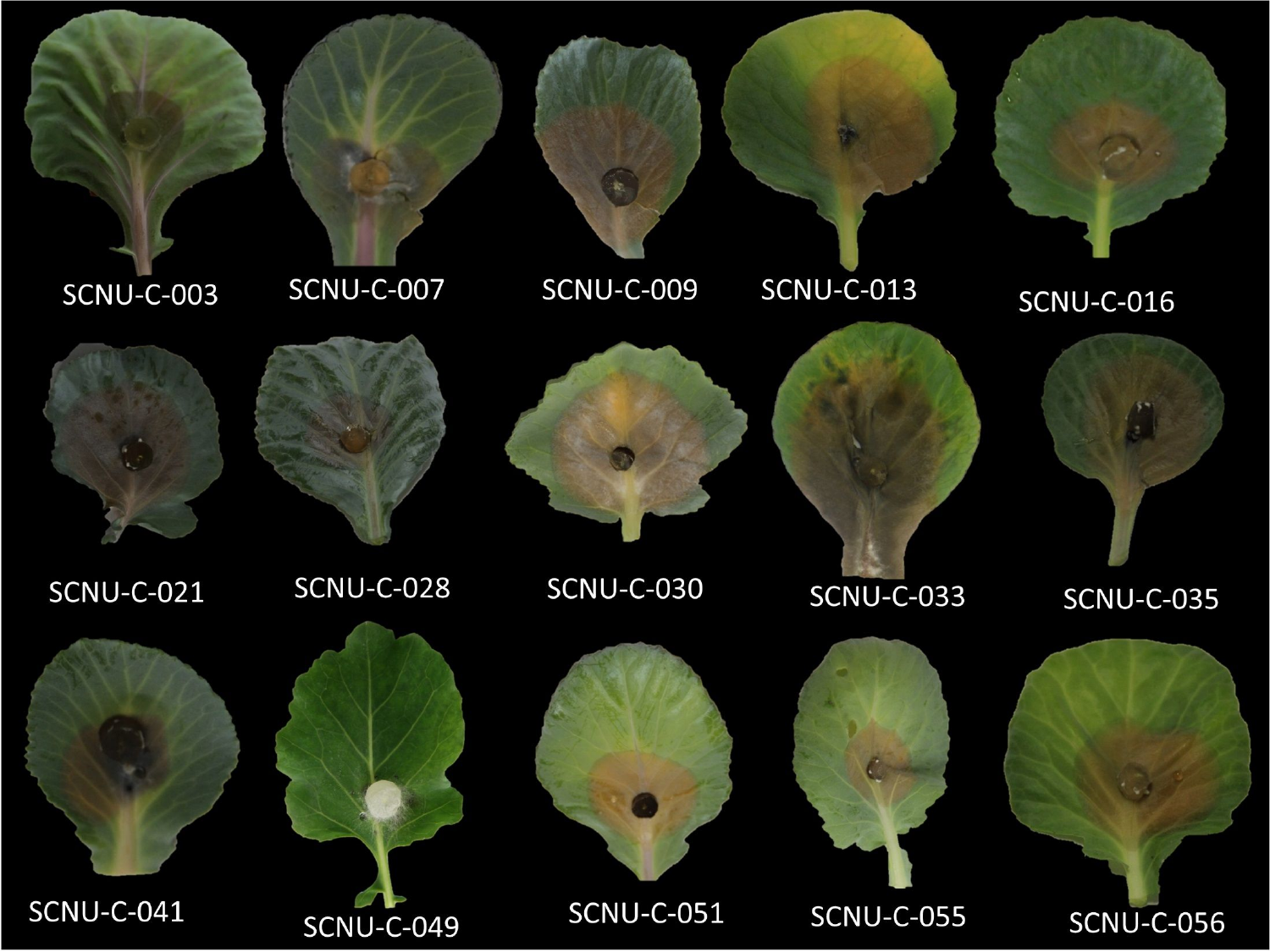

## Slide 2
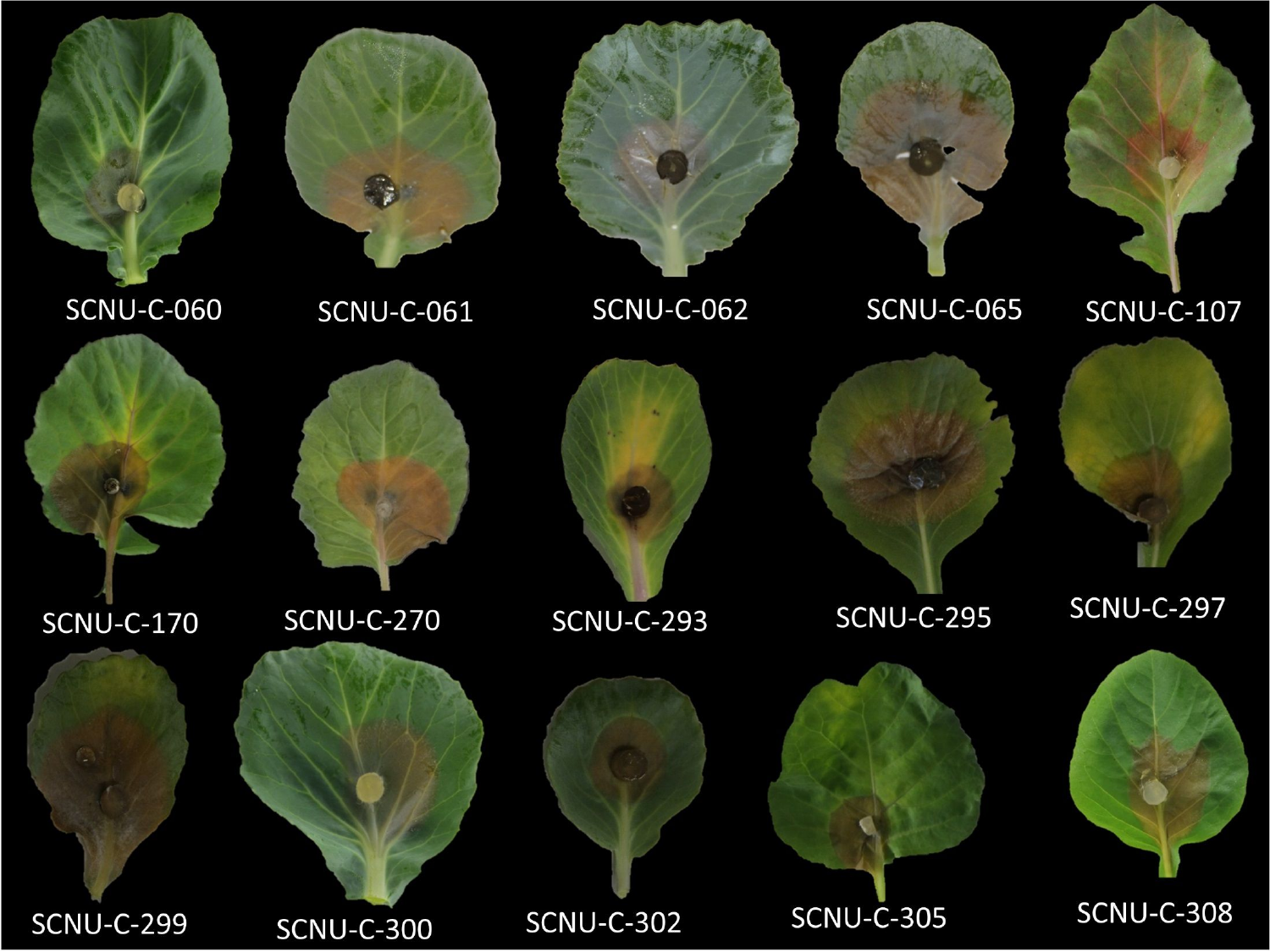

## Slide 3
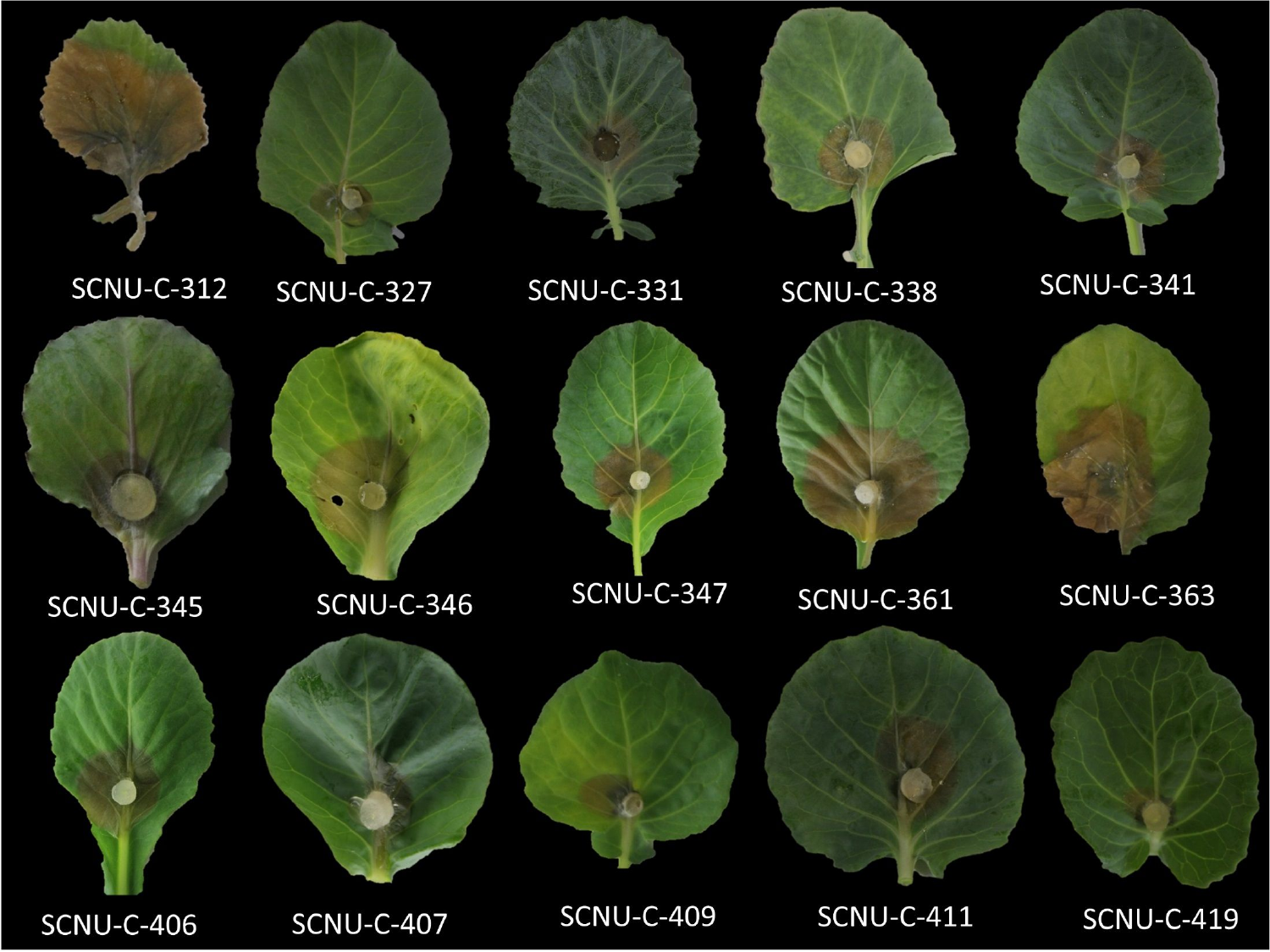

## Slide 4
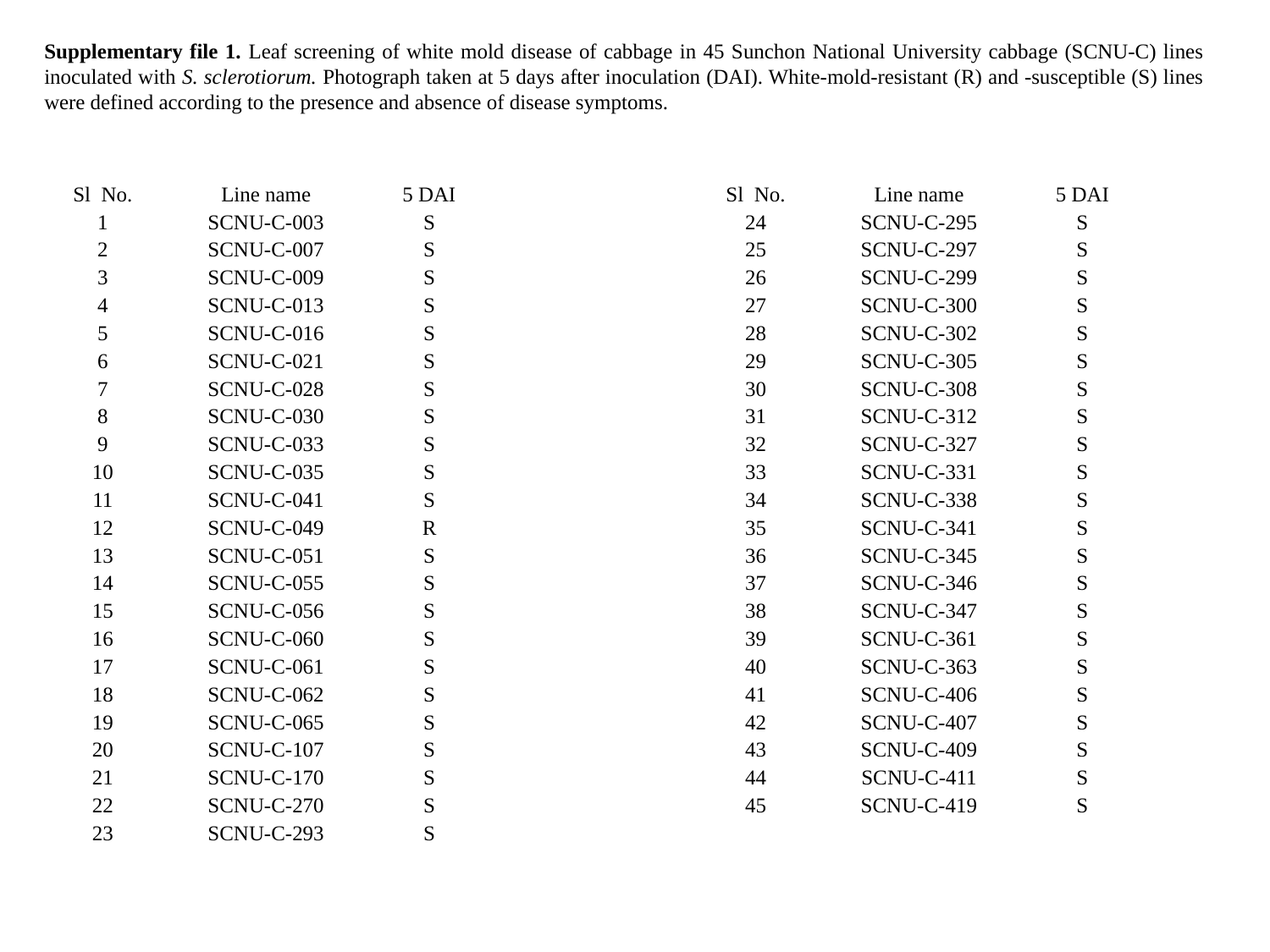

Supplementary file 1. Leaf screening of white mold disease of cabbage in 45 Sunchon National University cabbage (SCNU-C) lines inoculated with S. sclerotiorum. Photograph taken at 5 days after inoculation (DAI). White-mold-resistant (R) and -susceptible (S) lines were defined according to the presence and absence of disease symptoms.
| Sl No. | Line name | 5 DAI | | Sl No. | Line name | 5 DAI |
| --- | --- | --- | --- | --- | --- | --- |
| 1 | SCNU-C-003 | S | | 24 | SCNU-C-295 | S |
| 2 | SCNU-C-007 | S | | 25 | SCNU-C-297 | S |
| 3 | SCNU-C-009 | S | | 26 | SCNU-C-299 | S |
| 4 | SCNU-C-013 | S | | 27 | SCNU-C-300 | S |
| 5 | SCNU-C-016 | S | | 28 | SCNU-C-302 | S |
| 6 | SCNU-C-021 | S | | 29 | SCNU-C-305 | S |
| 7 | SCNU-C-028 | S | | 30 | SCNU-C-308 | S |
| 8 | SCNU-C-030 | S | | 31 | SCNU-C-312 | S |
| 9 | SCNU-C-033 | S | | 32 | SCNU-C-327 | S |
| 10 | SCNU-C-035 | S | | 33 | SCNU-C-331 | S |
| 11 | SCNU-C-041 | S | | 34 | SCNU-C-338 | S |
| 12 | SCNU-C-049 | R | | 35 | SCNU-C-341 | S |
| 13 | SCNU-C-051 | S | | 36 | SCNU-C-345 | S |
| 14 | SCNU-C-055 | S | | 37 | SCNU-C-346 | S |
| 15 | SCNU-C-056 | S | | 38 | SCNU-C-347 | S |
| 16 | SCNU-C-060 | S | | 39 | SCNU-C-361 | S |
| 17 | SCNU-C-061 | S | | 40 | SCNU-C-363 | S |
| 18 | SCNU-C-062 | S | | 41 | SCNU-C-406 | S |
| 19 | SCNU-C-065 | S | | 42 | SCNU-C-407 | S |
| 20 | SCNU-C-107 | S | | 43 | SCNU-C-409 | S |
| 21 | SCNU-C-170 | S | | 44 | SCNU-C-411 | S |
| 22 | SCNU-C-270 | S | | 45 | SCNU-C-419 | S |
| 23 | SCNU-C-293 | S | | | | |
