# Supplementary material for: Glucosinolate Profiling and Expression Analysis of Glucosinolate Biosynthesis Genes Differentiate White Mold Resistant and Susceptible Cabbage Lines
Source: Int J Mol Sci. 2018 Dec 13;19(12):4037. doi: 10.3390/ijms19124037 (PMC6321582; doi:10.3390/ijms19124037)
Supplement: Supplementary file 1 [file ijms-19-04037-s001.zip › Supplementary file 2.pptx]

## Slide 1
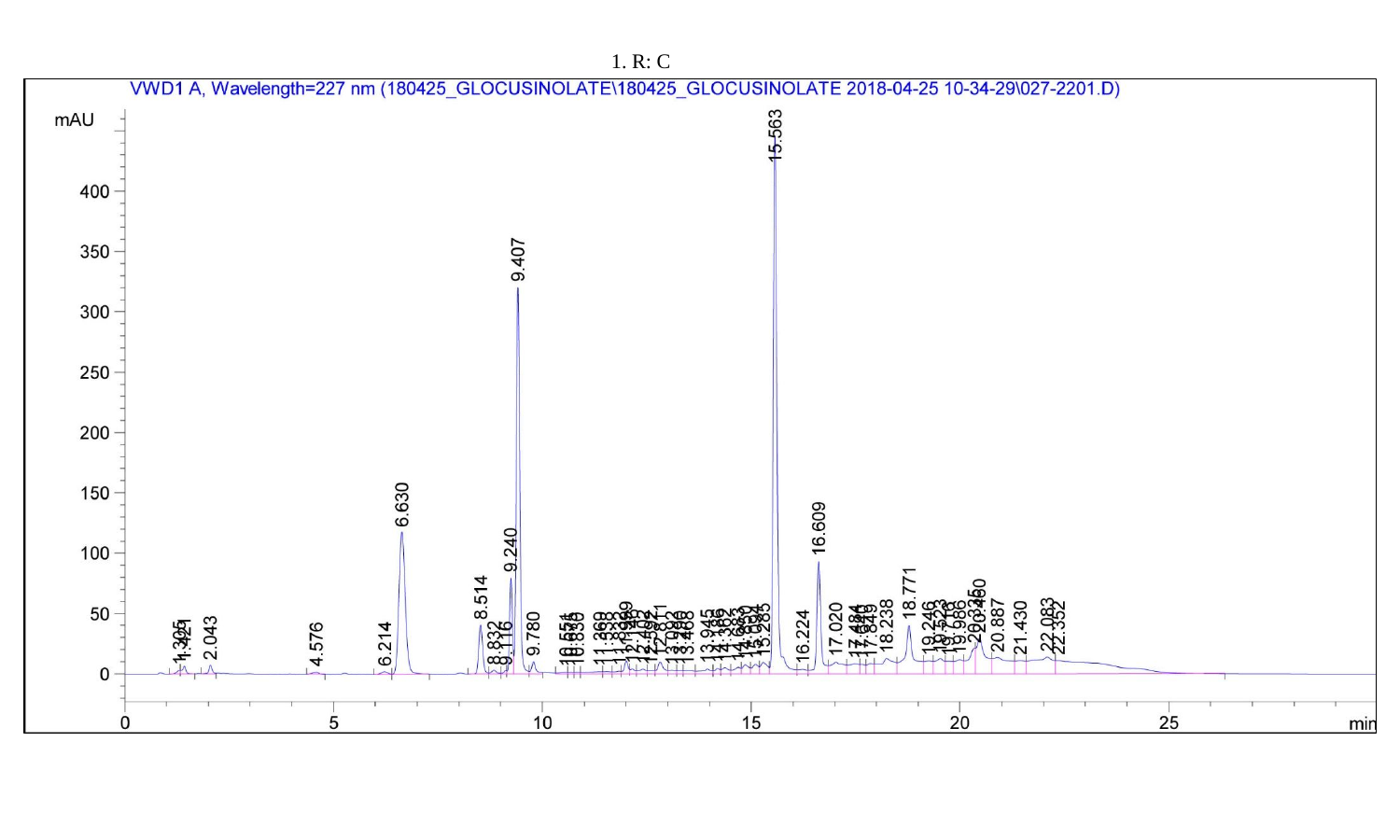

1. R: C

## Slide 2
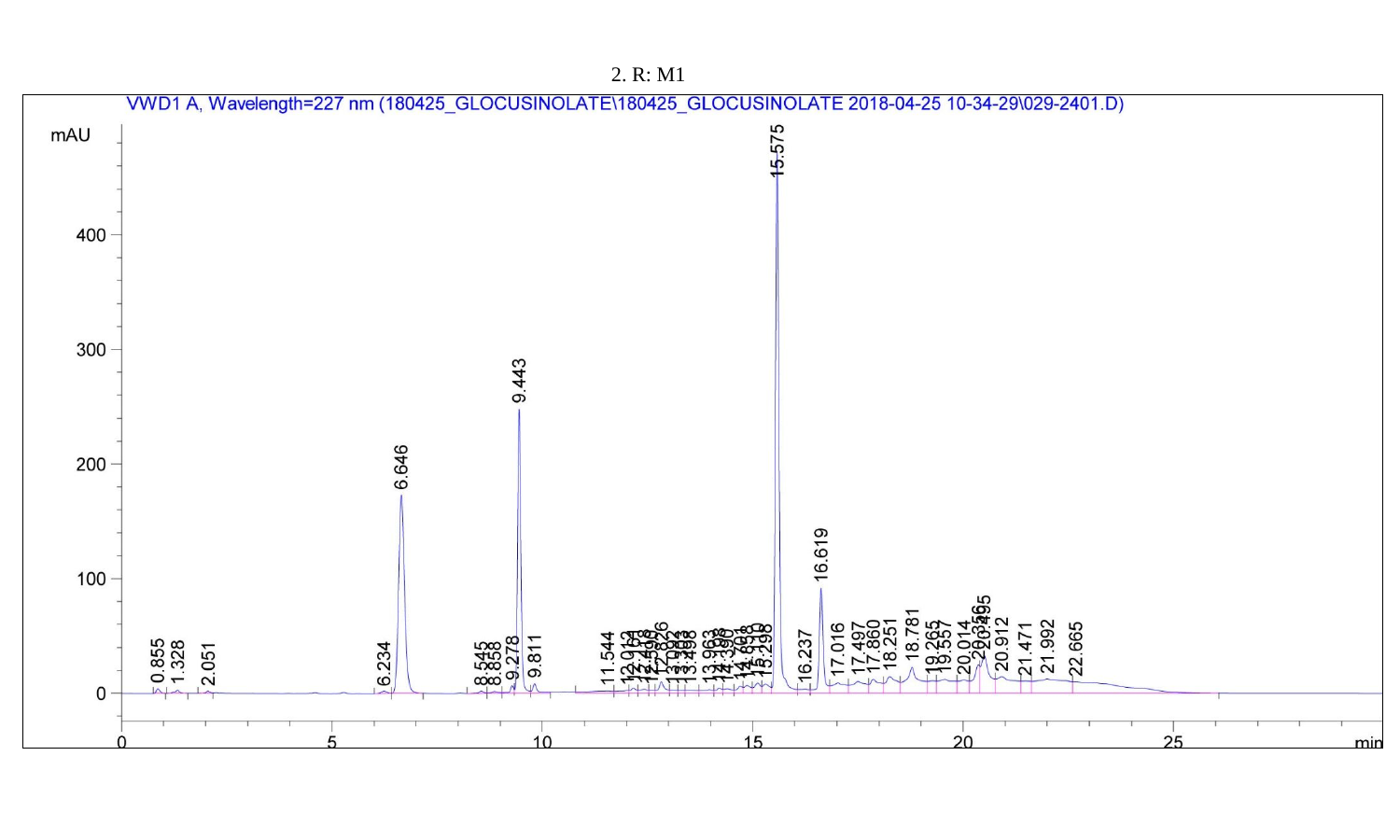

2. R: M1

## Slide 3
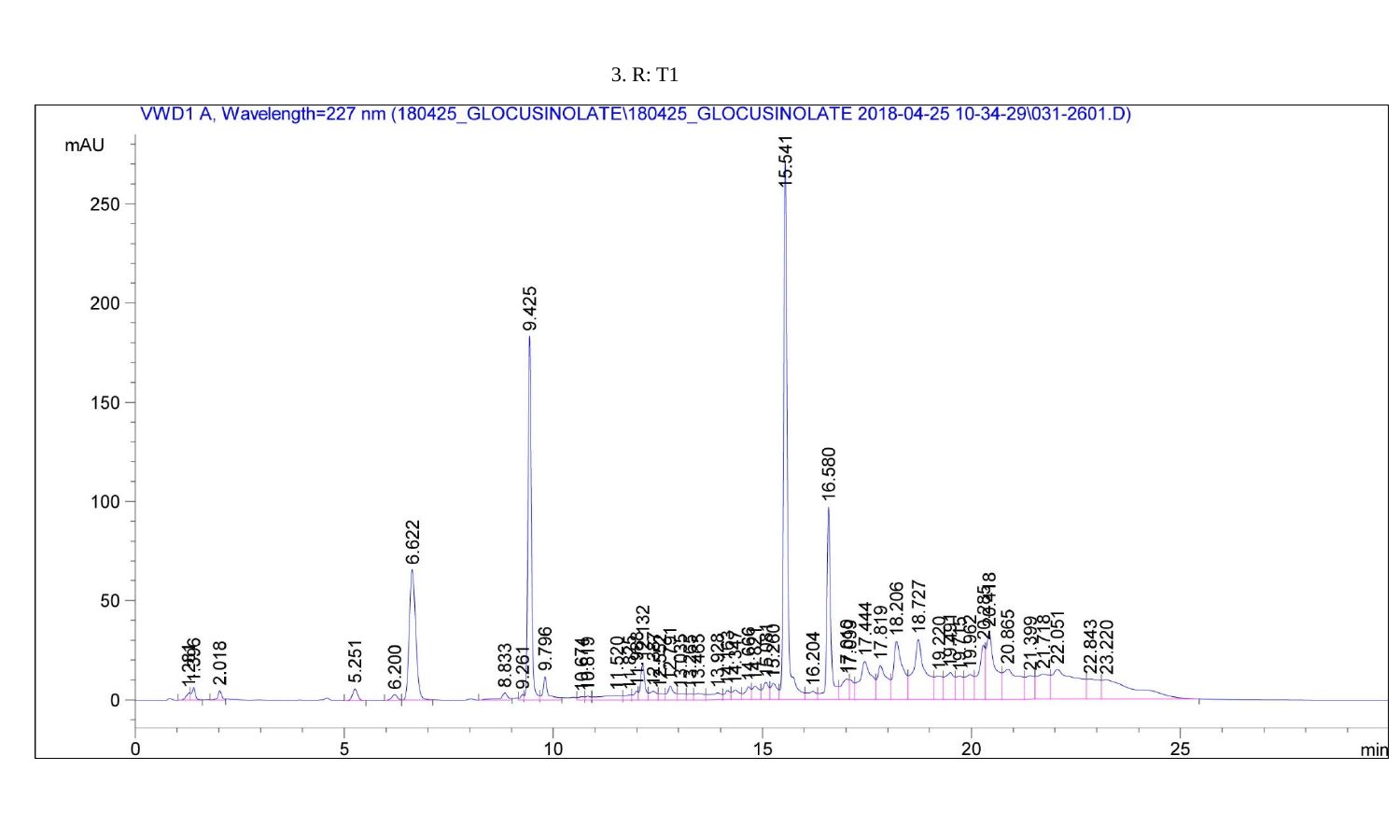

3. R: T1

## Slide 4
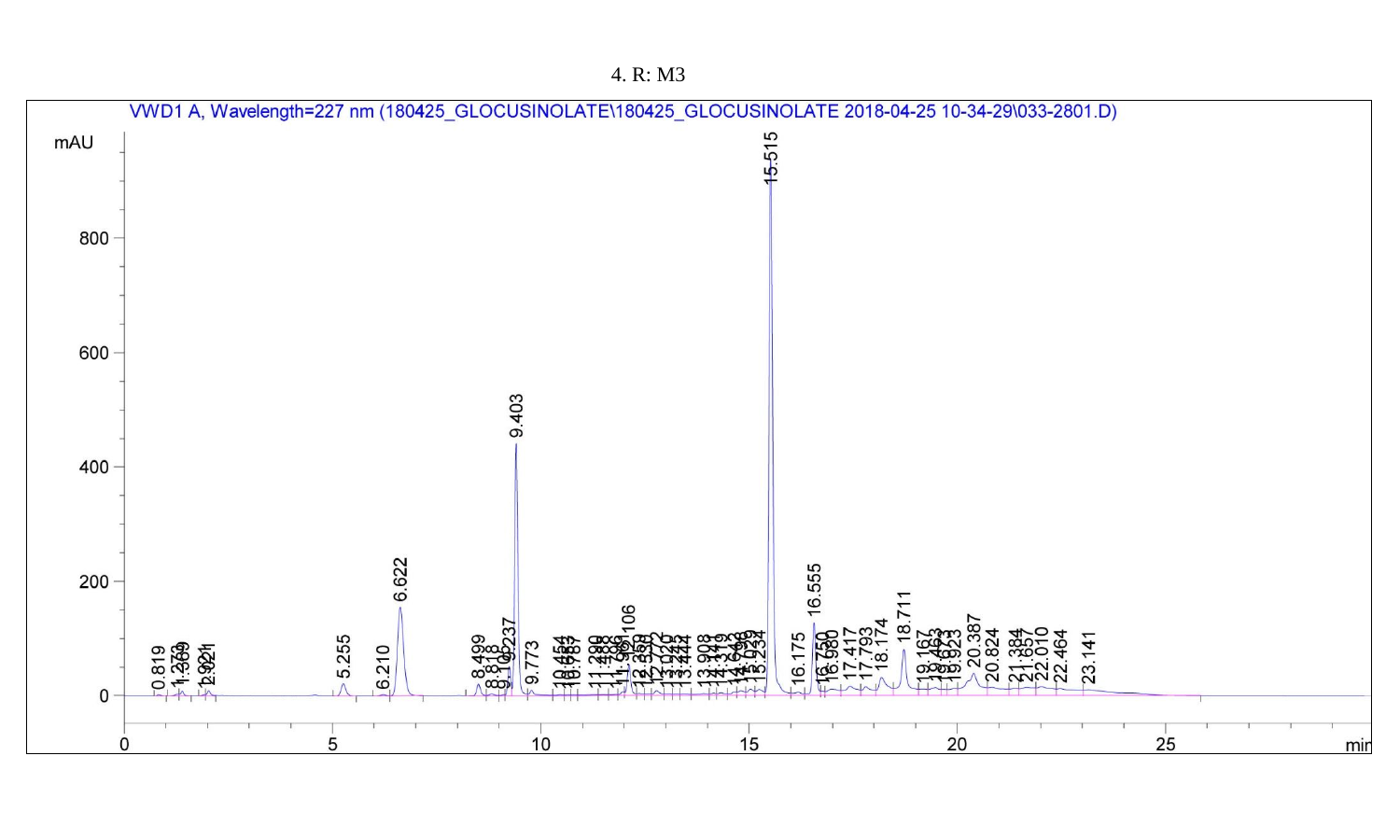

4. R: M3

## Slide 5
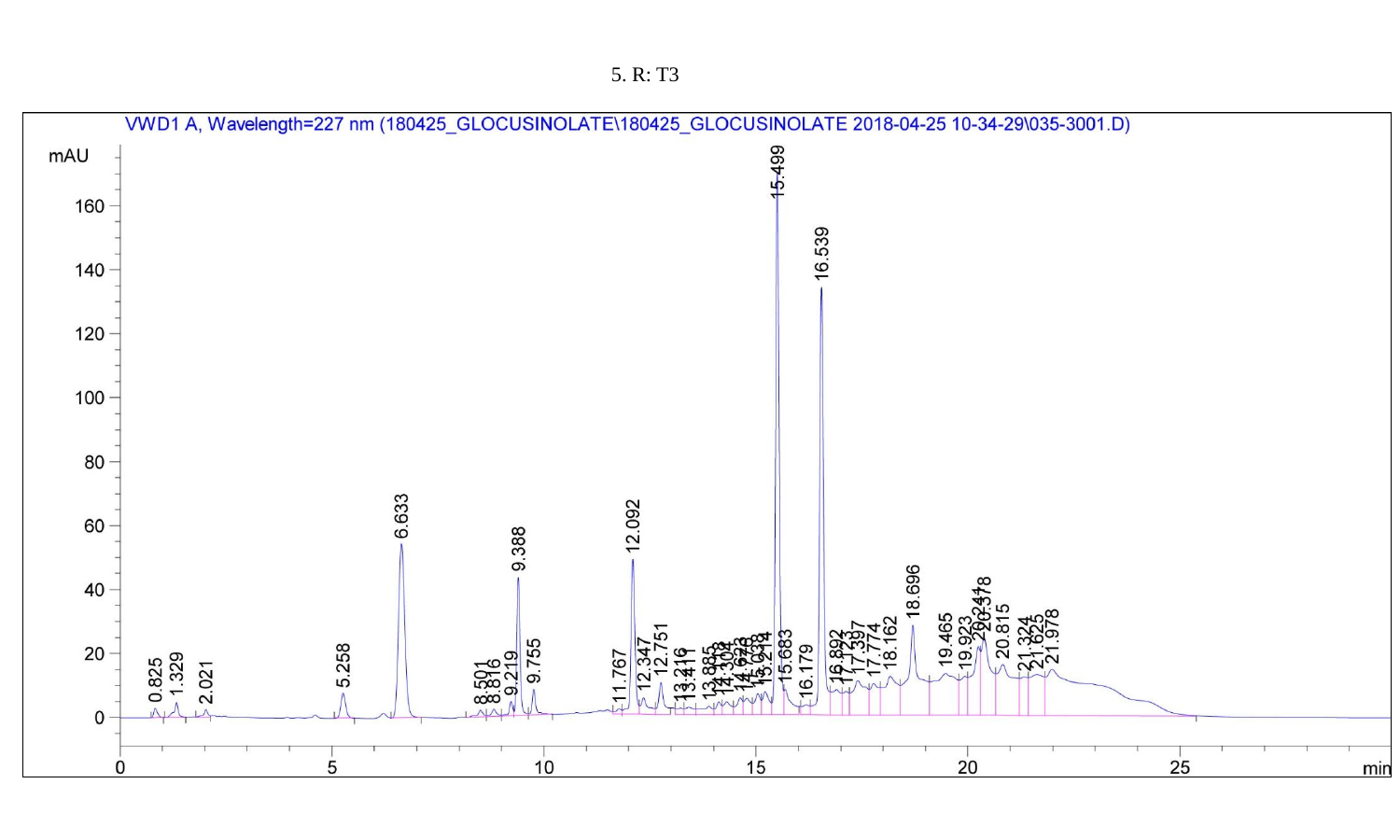

5. R: T3

## Slide 6
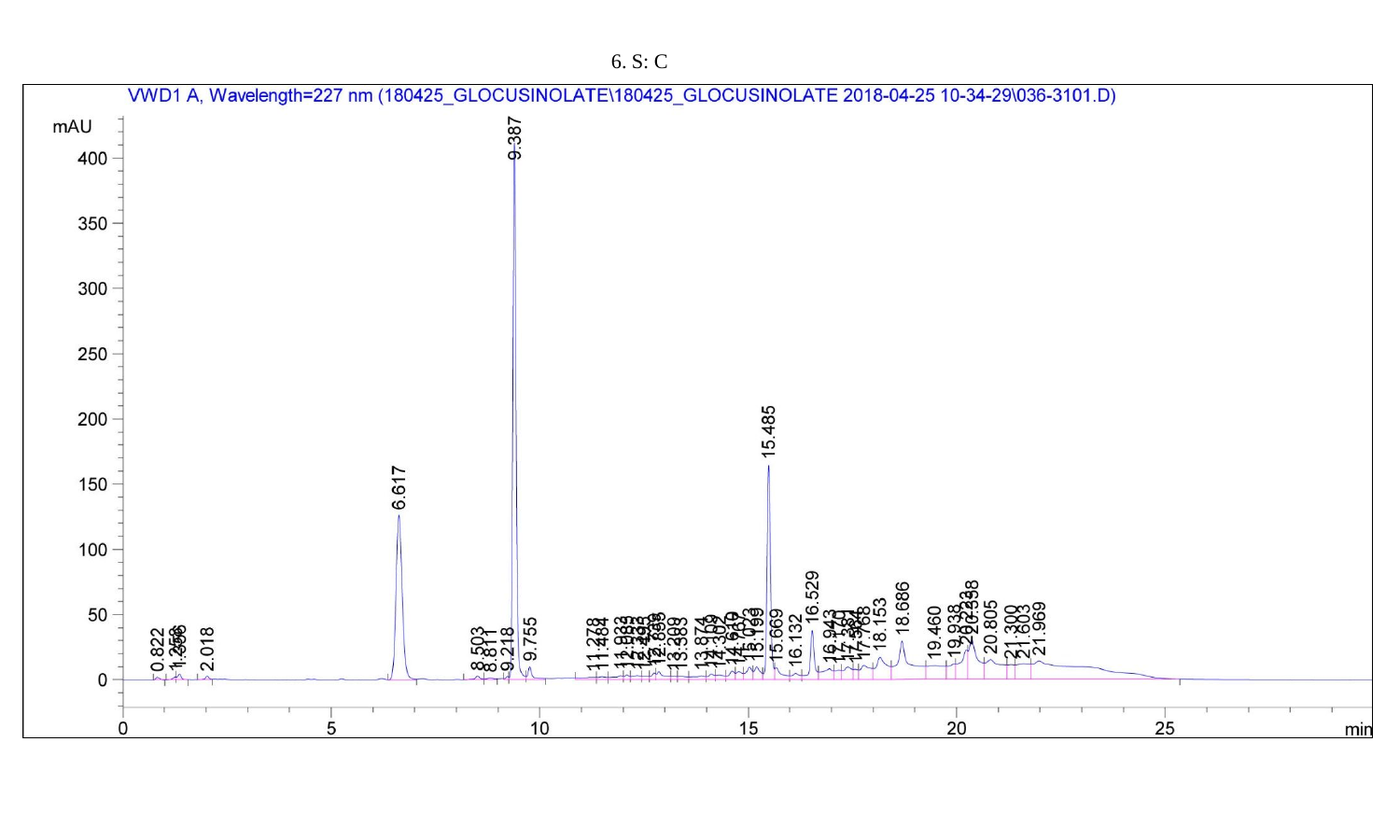

6. S: C

## Slide 7
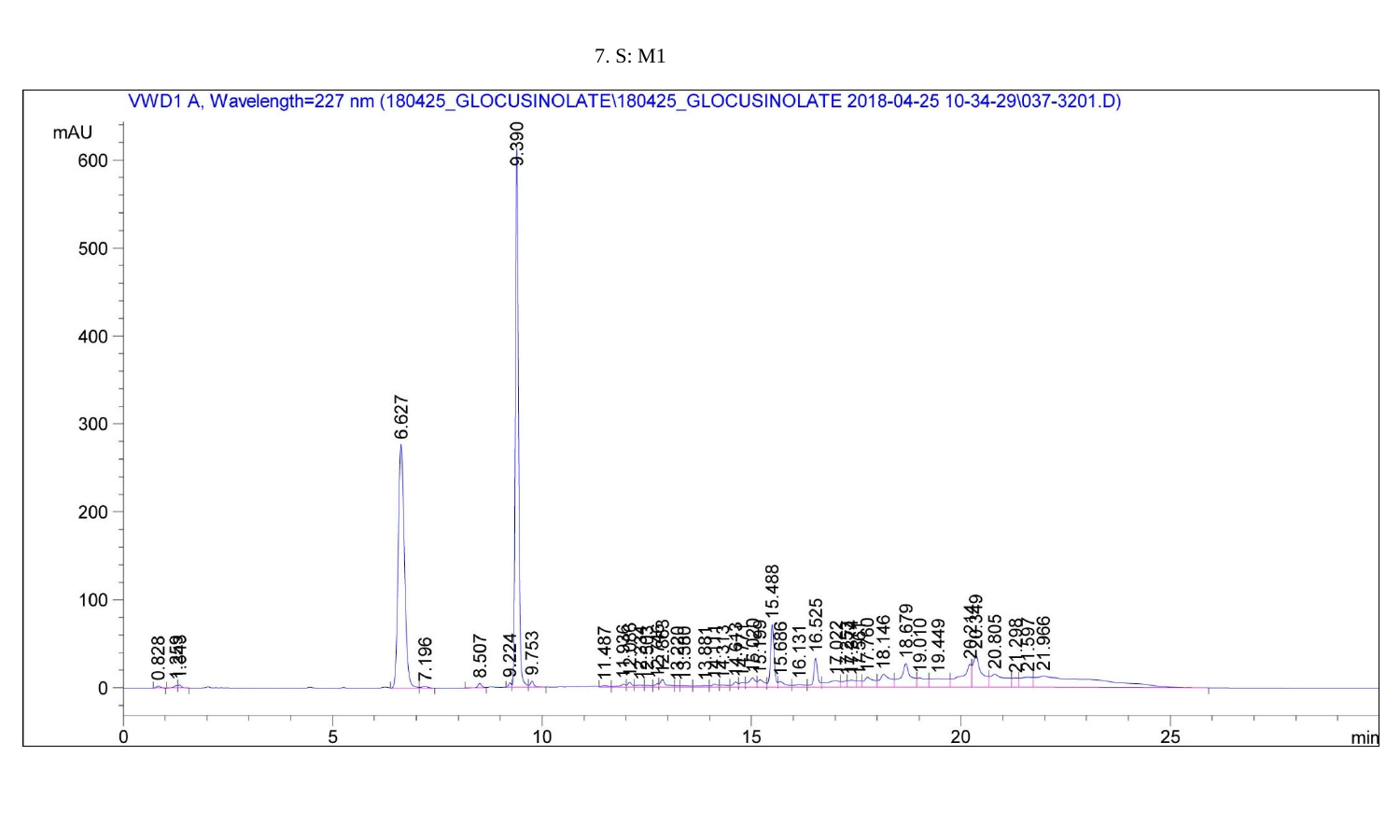

7. S: M1

## Slide 8
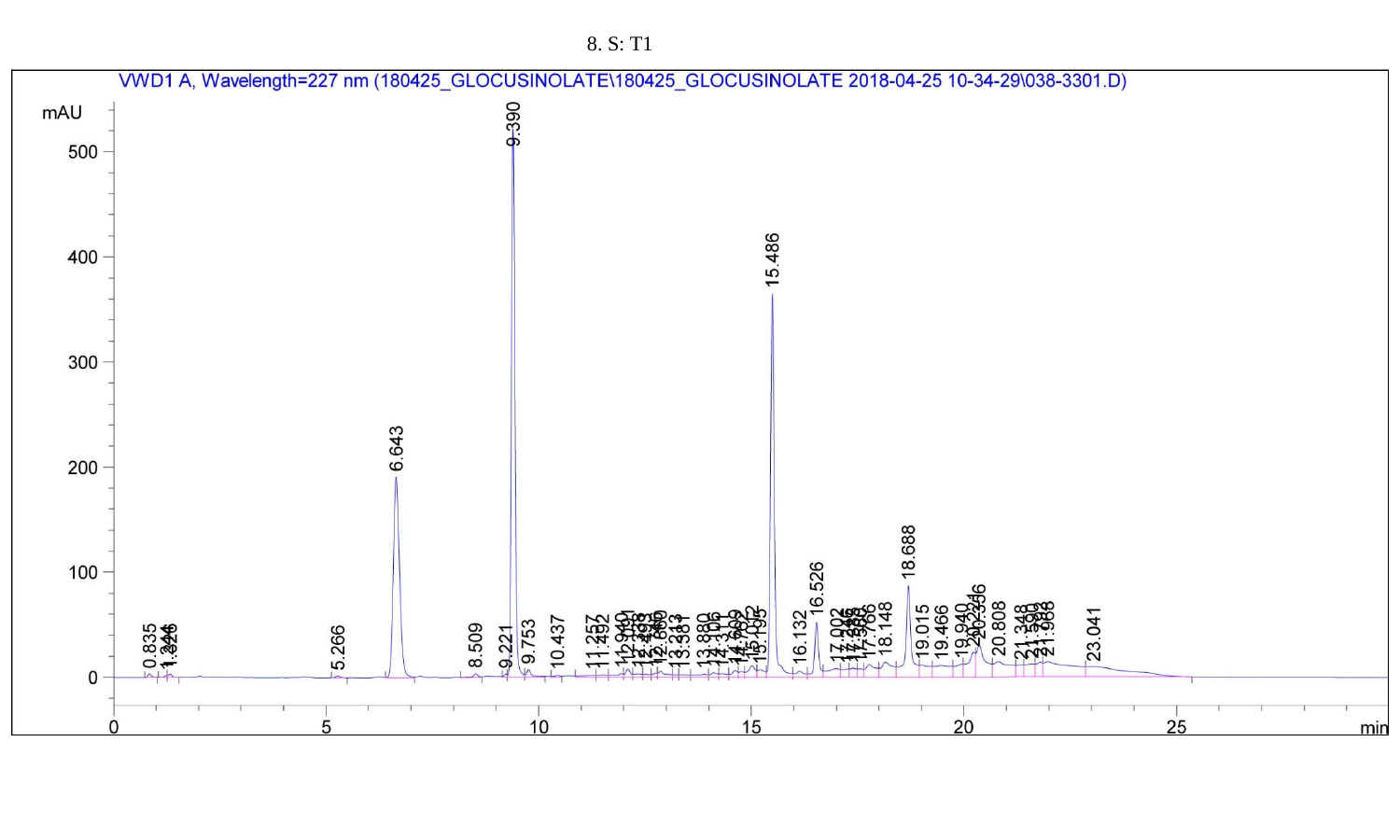

8. S: T1

## Slide 9
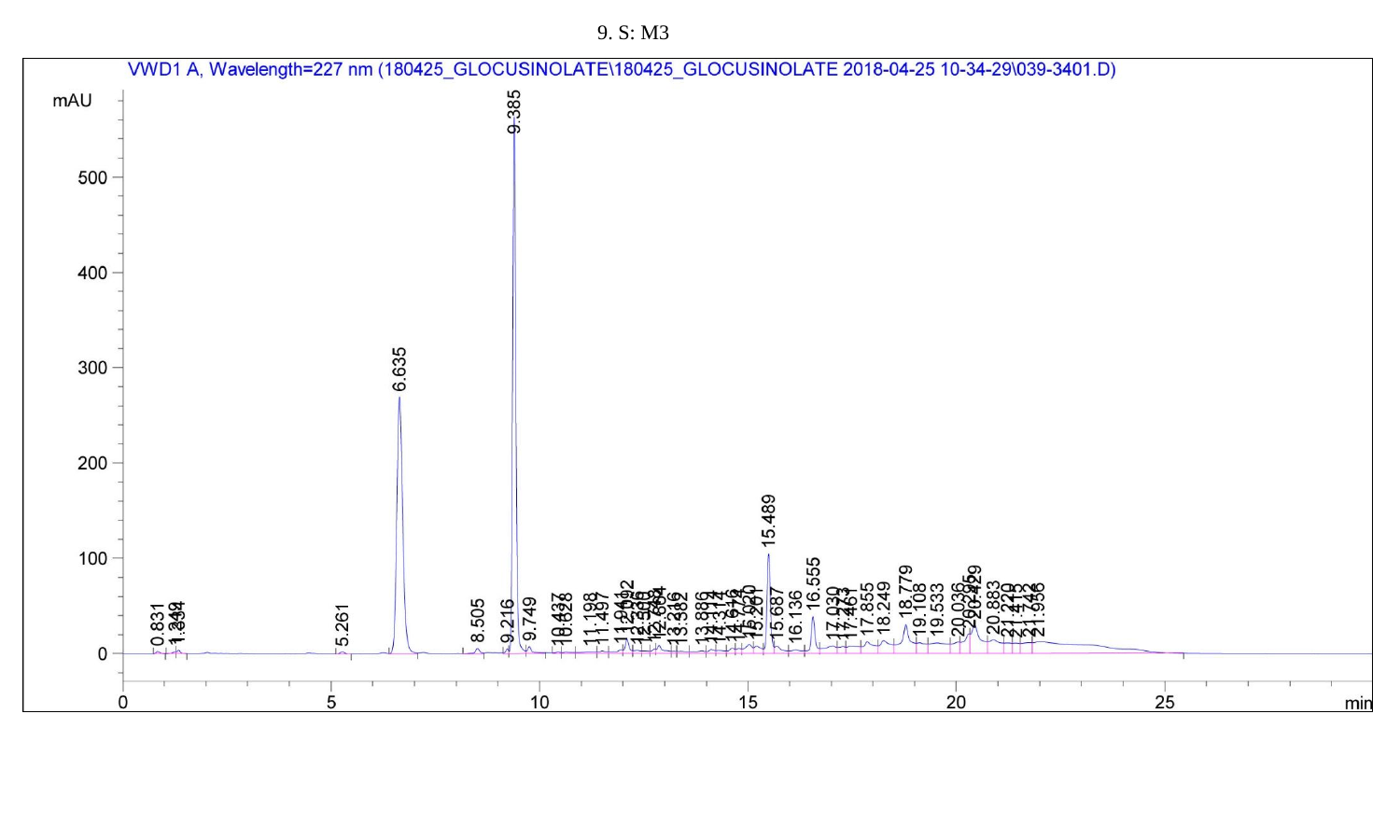

9. S: M3

## Slide 10
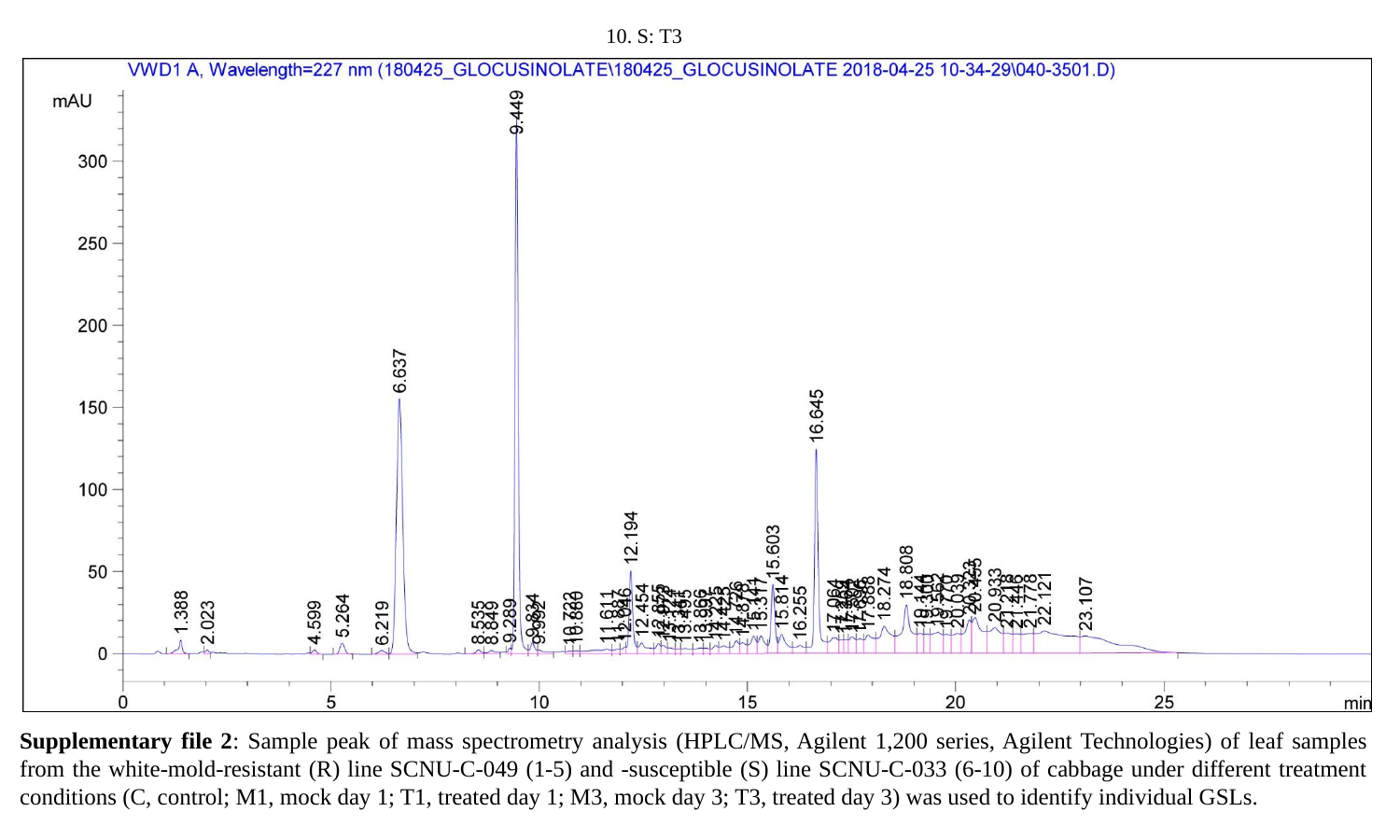

10. S: T3
Supplementary file 2: Sample peak of mass spectrometry analysis (HPLC/MS, Agilent 1,200 series, Agilent Technologies) of leaf samples from the white-mold-resistant (R) line SCNU-C-049 (1-5) and -susceptible (S) line SCNU-C-033 (6-10) of cabbage under different treatment conditions (C, control; M1, mock day 1; T1, treated day 1; M3, mock day 3; T3, treated day 3) was used to identify individual GSLs.
