# Supplementary material for: Glucosinolate Profiling and Expression Analysis of Glucosinolate Biosynthesis Genes Differentiate White Mold Resistant and Susceptible Cabbage Lines
Source: Int J Mol Sci. 2018 Dec 13;19(12):4037. doi: 10.3390/ijms19124037 (PMC6321582; doi:10.3390/ijms19124037)
Supplement: Supplementary file 1 [file ijms-19-04037-s001.zip › Supplementary file 4.pptx]

## Slide 1
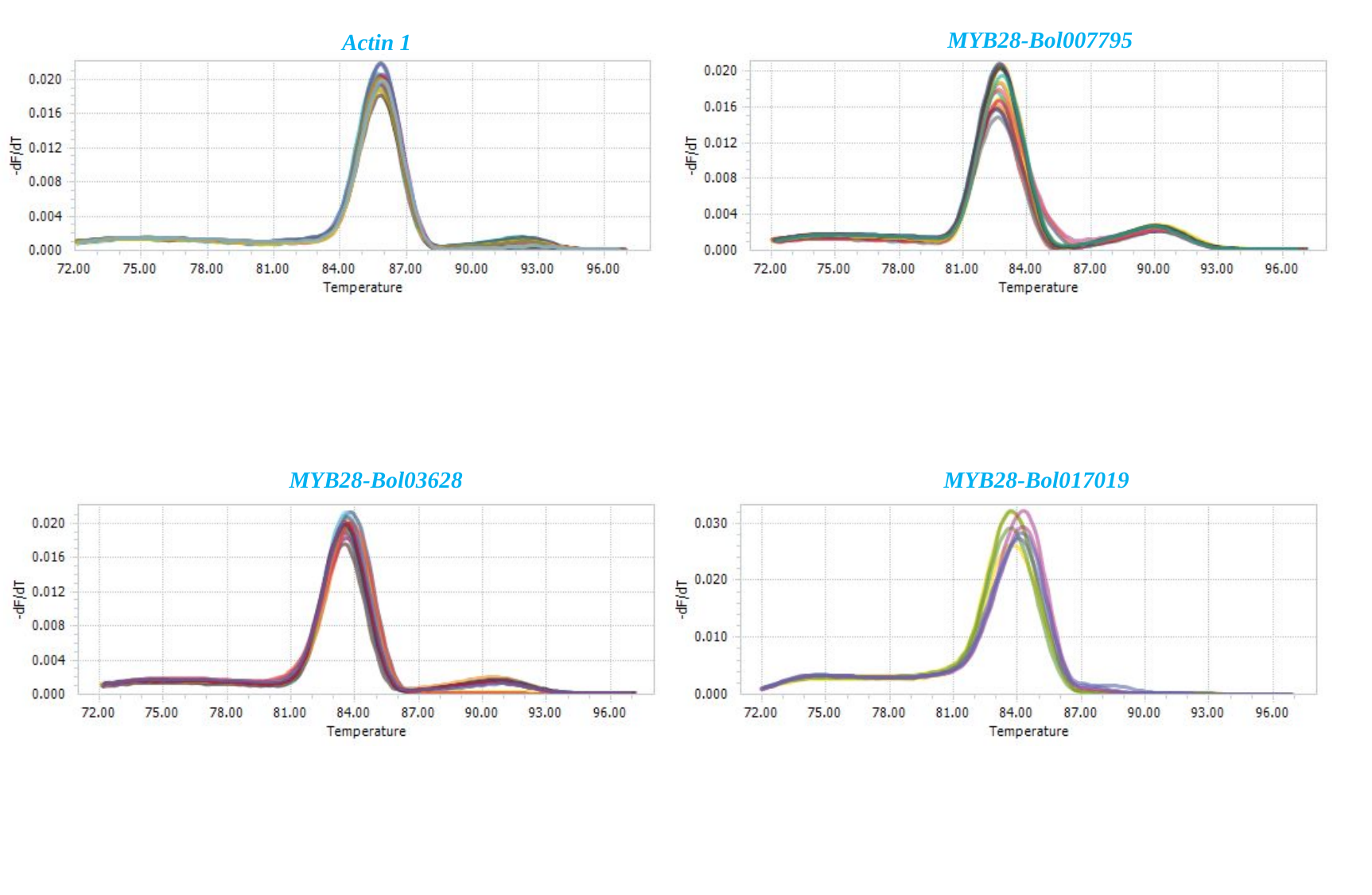

MYB28-Bol007795
Actin 1
MYB28-Bol017019
MYB28-Bol03628

## Slide 2
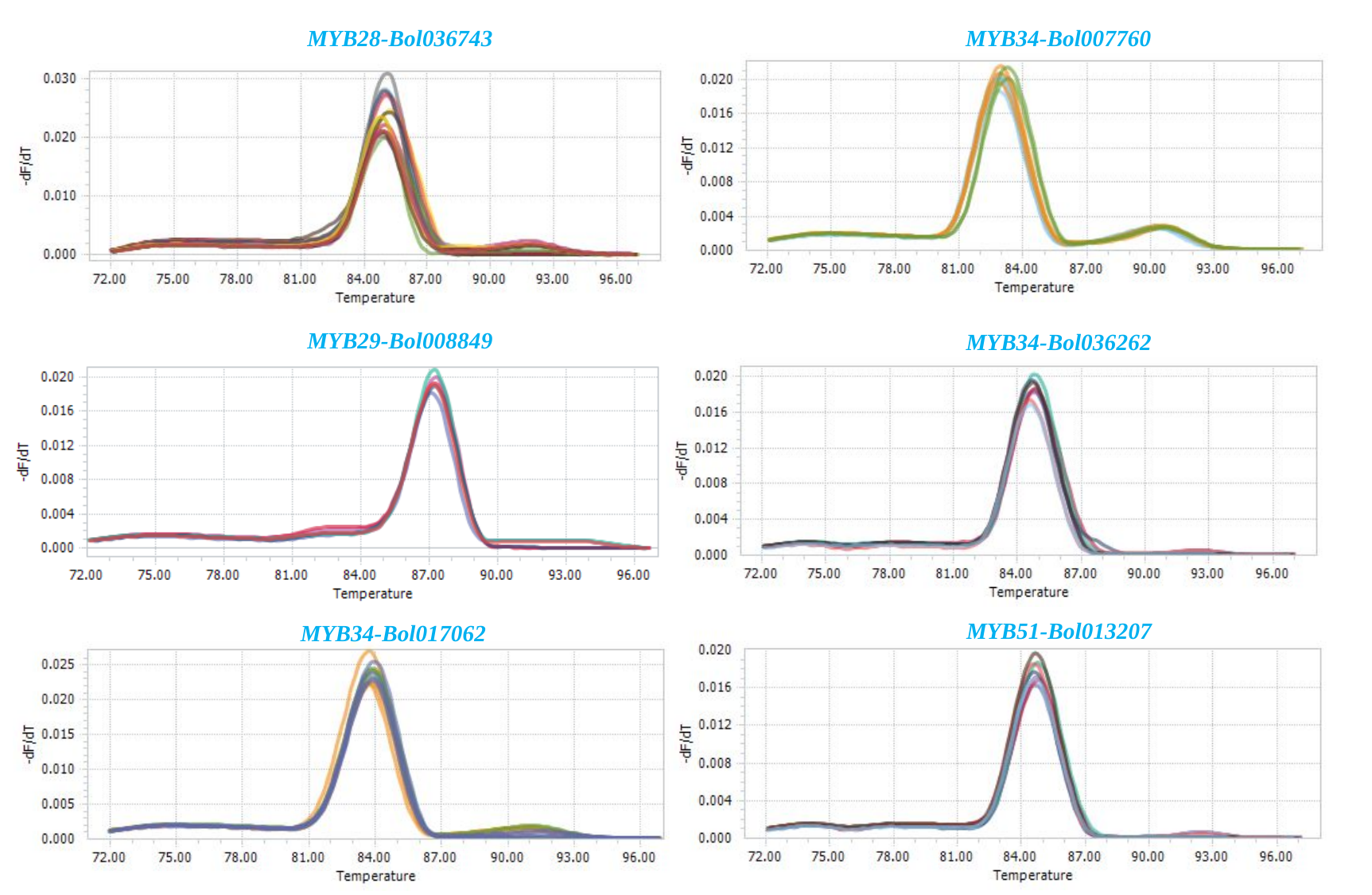

MYB28-Bol036743
MYB34-Bol007760
MYB29-Bol008849
MYB34-Bol036262
MYB51-Bol013207
MYB34-Bol017062

## Slide 3
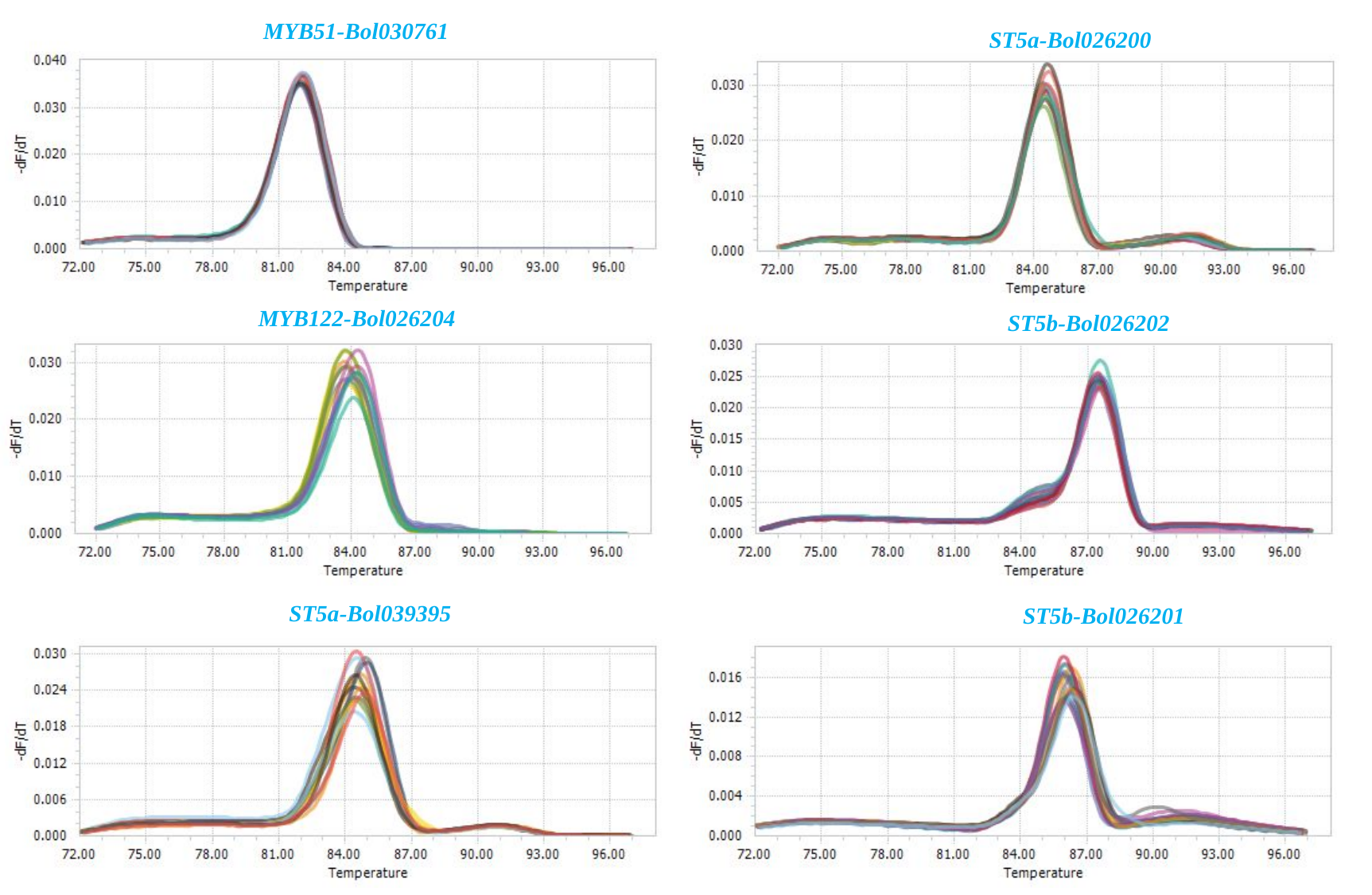

MYB51-Bol030761
ST5a-Bol026200
MYB122-Bol026204
ST5b-Bol026202
ST5a-Bol039395
ST5b-Bol026201

## Slide 4
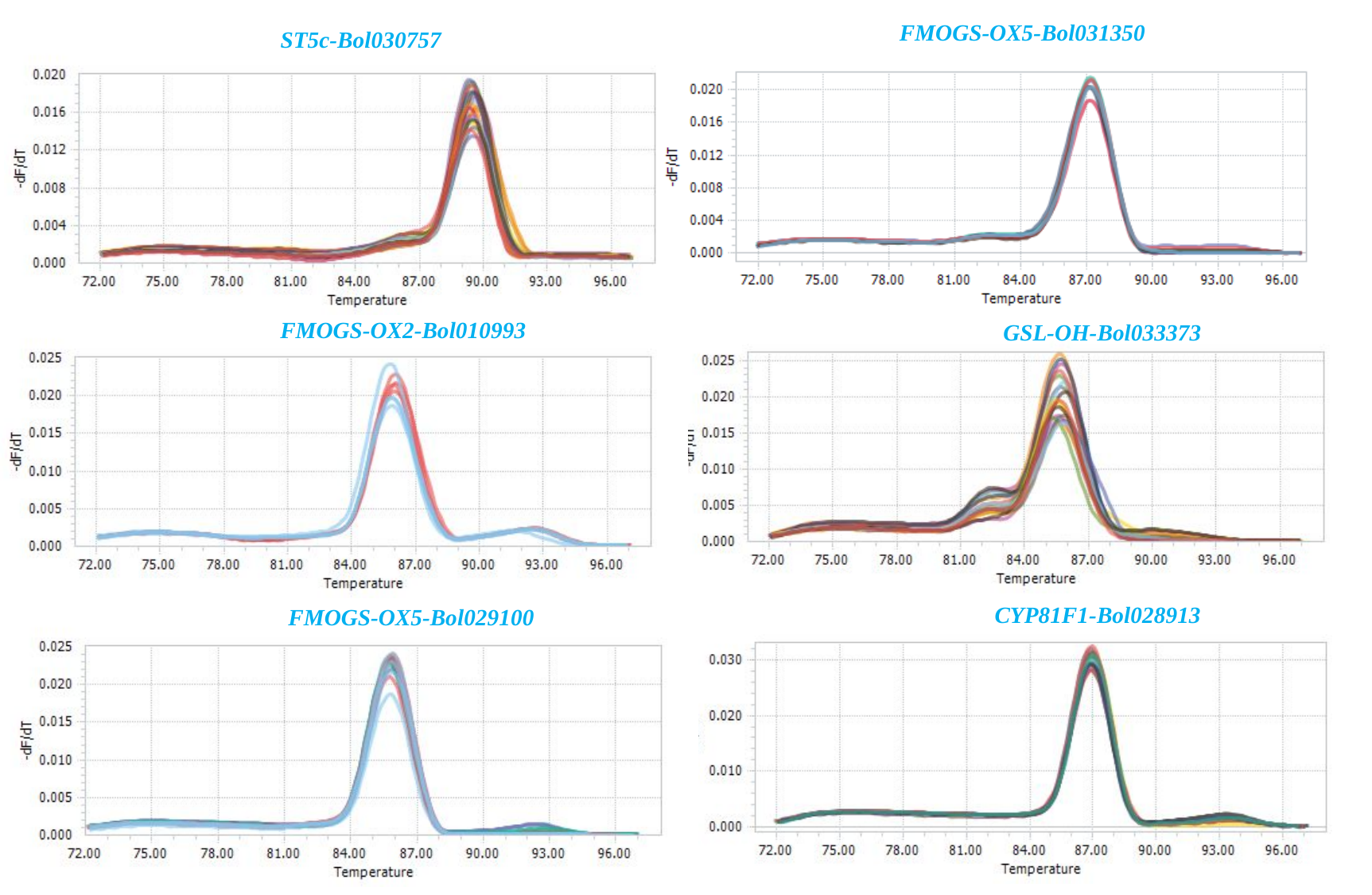

FMOGS-OX5-Bol031350
ST5c-Bol030757
FMOGS-OX2-Bol010993
GSL-OH-Bol033373
CYP81F1-Bol028913
FMOGS-OX5-Bol029100

## Slide 5
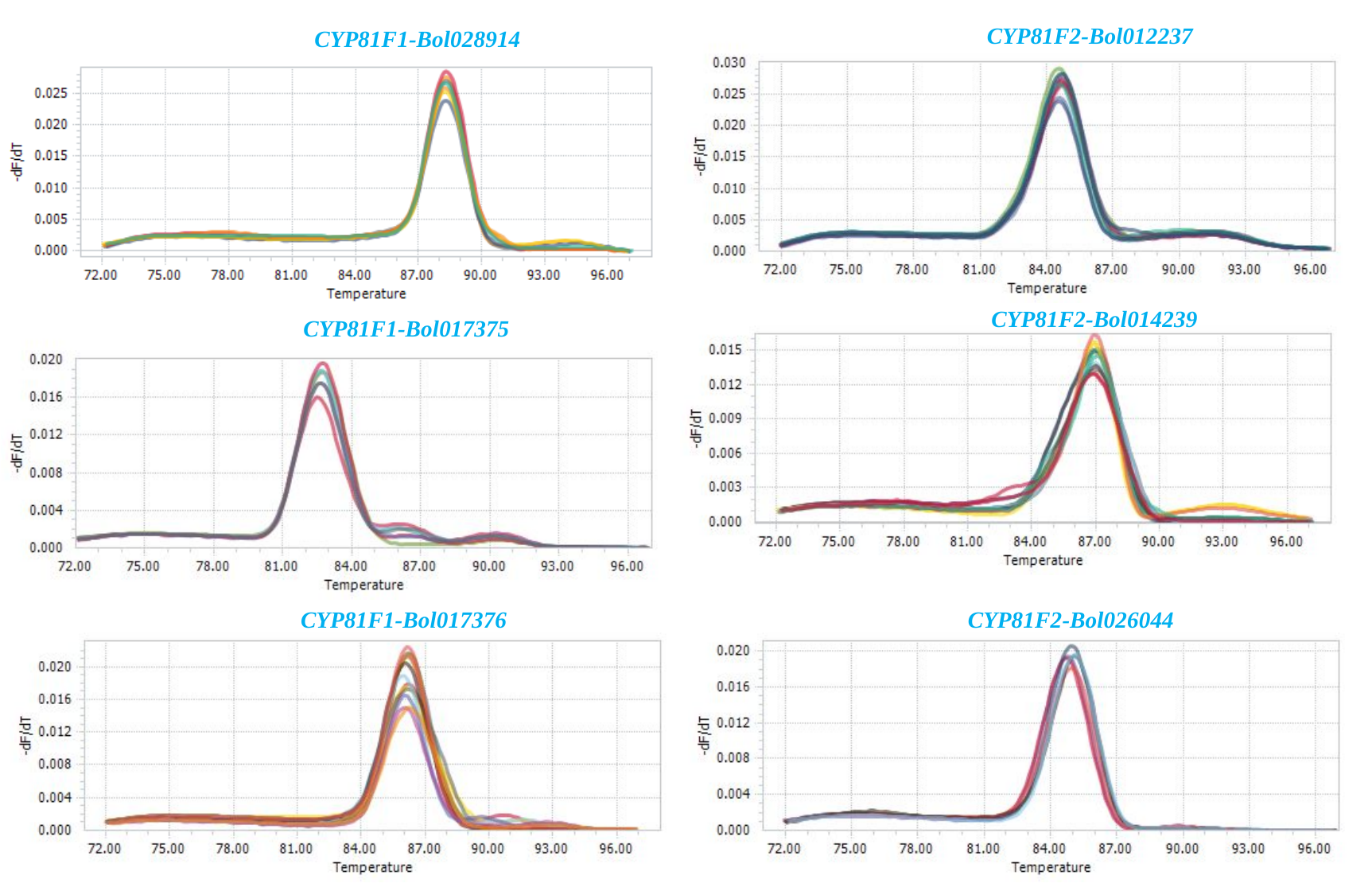

CYP81F2-Bol012237
CYP81F1-Bol028914
CYP81F2-Bol014239
CYP81F1-Bol017375
CYP81F2-Bol026044
CYP81F1-Bol017376

## Slide 6
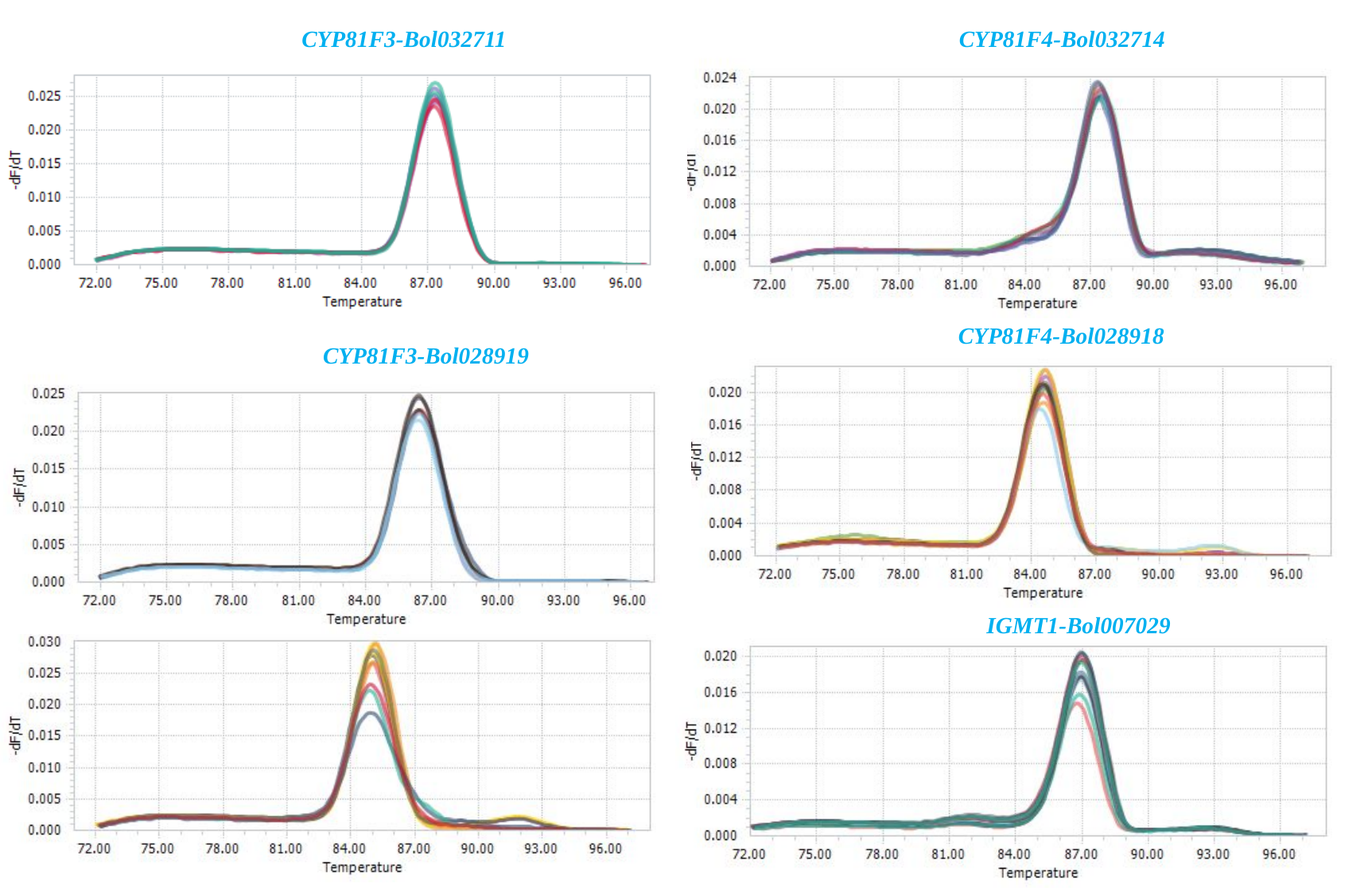

CYP81F3-Bol032711
CYP81F4-Bol032714
CYP81F4-Bol028918
CYP81F3-Bol028919
CYP81F4-Bol032712
IGMT1-Bol007029

## Slide 7
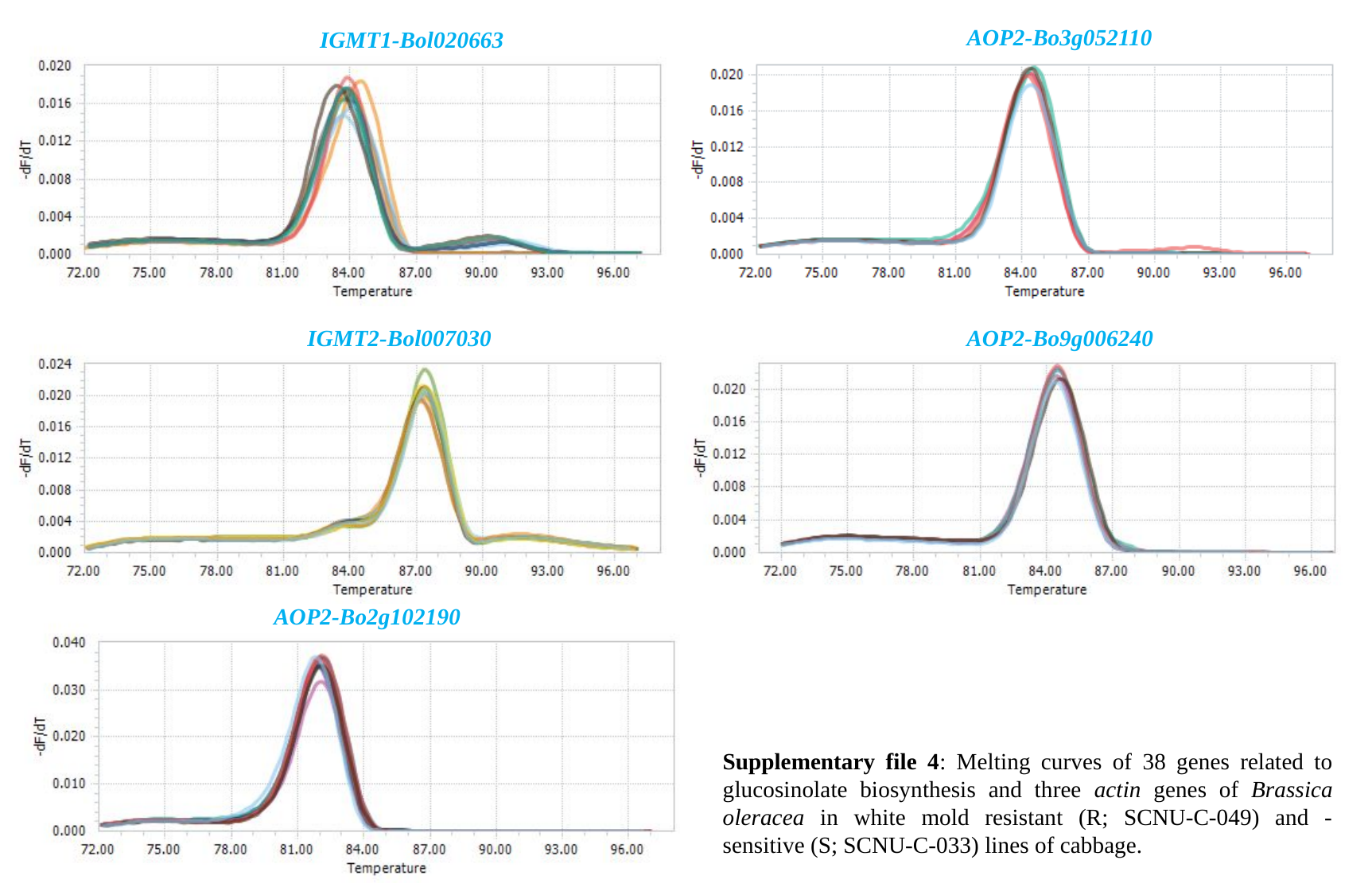

AOP2-Bo3g052110
IGMT1-Bol020663
IGMT2-Bol007030
AOP2-Bo9g006240
AOP2-Bo2g102190
Supplementary file 4: Melting curves of 38 genes related to glucosinolate biosynthesis and three actin genes of Brassica oleracea in white mold resistant (R; SCNU-C-049) and -sensitive (S; SCNU-C-033) lines of cabbage.
