# Supplementary material for: Glucosinolate Profiling and Expression Analysis of Glucosinolate Biosynthesis Genes Differentiate White Mold Resistant and Susceptible Cabbage Lines
Source: Int J Mol Sci. 2018 Dec 13;19(12):4037. doi: 10.3390/ijms19124037 (PMC6321582; doi:10.3390/ijms19124037)
Supplement: Supplementary file 1 [file ijms-19-04037-s001.zip › Supplementary file 5.pptx]

## Slide 1
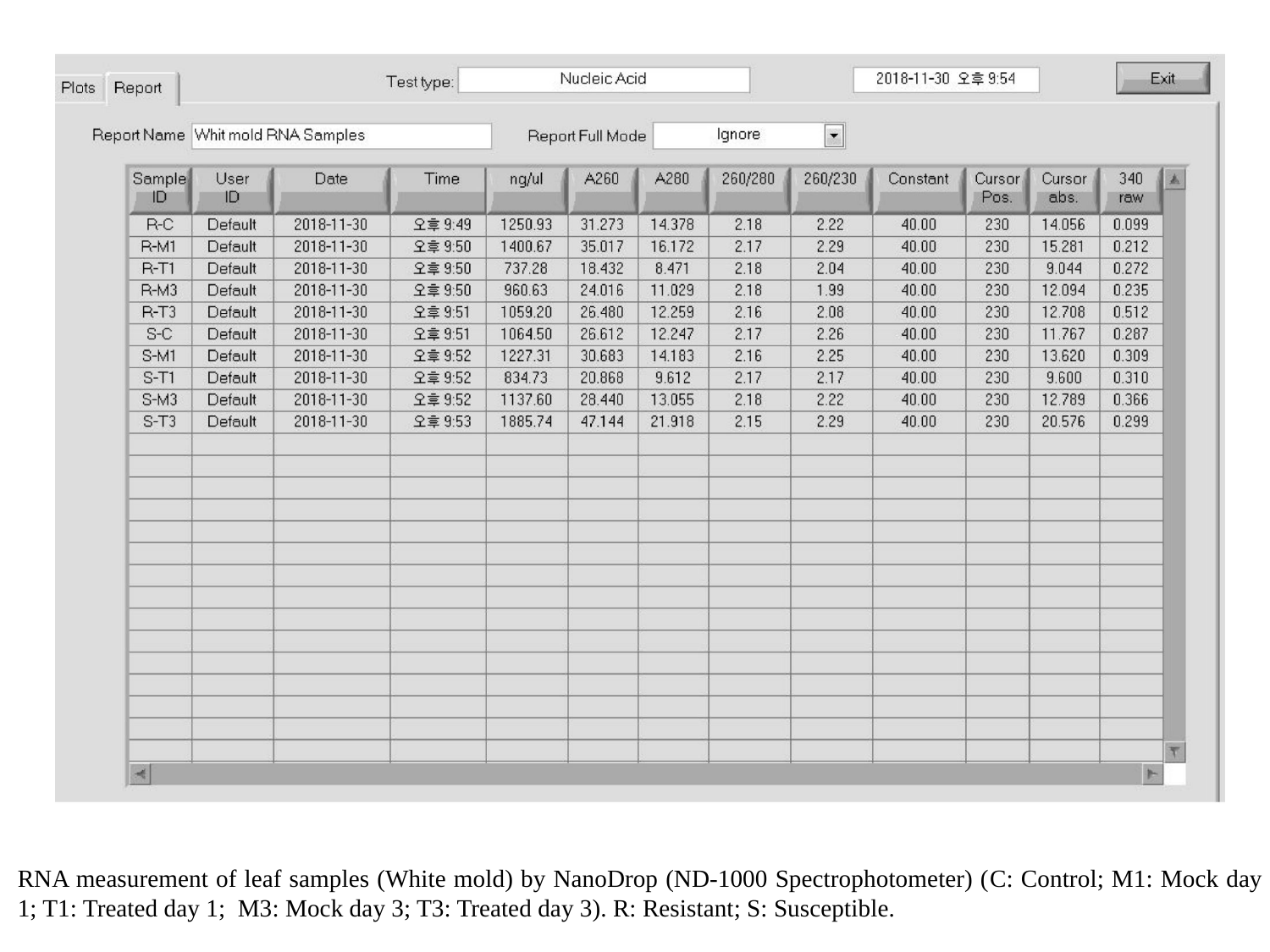

RNA measurement of leaf samples (White mold) by NanoDrop (ND-1000 Spectrophotometer) (C: Control; M1: Mock day 1; T1: Treated day 1; M3: Mock day 3; T3: Treated day 3). R: Resistant; S: Susceptible.

## Slide 2
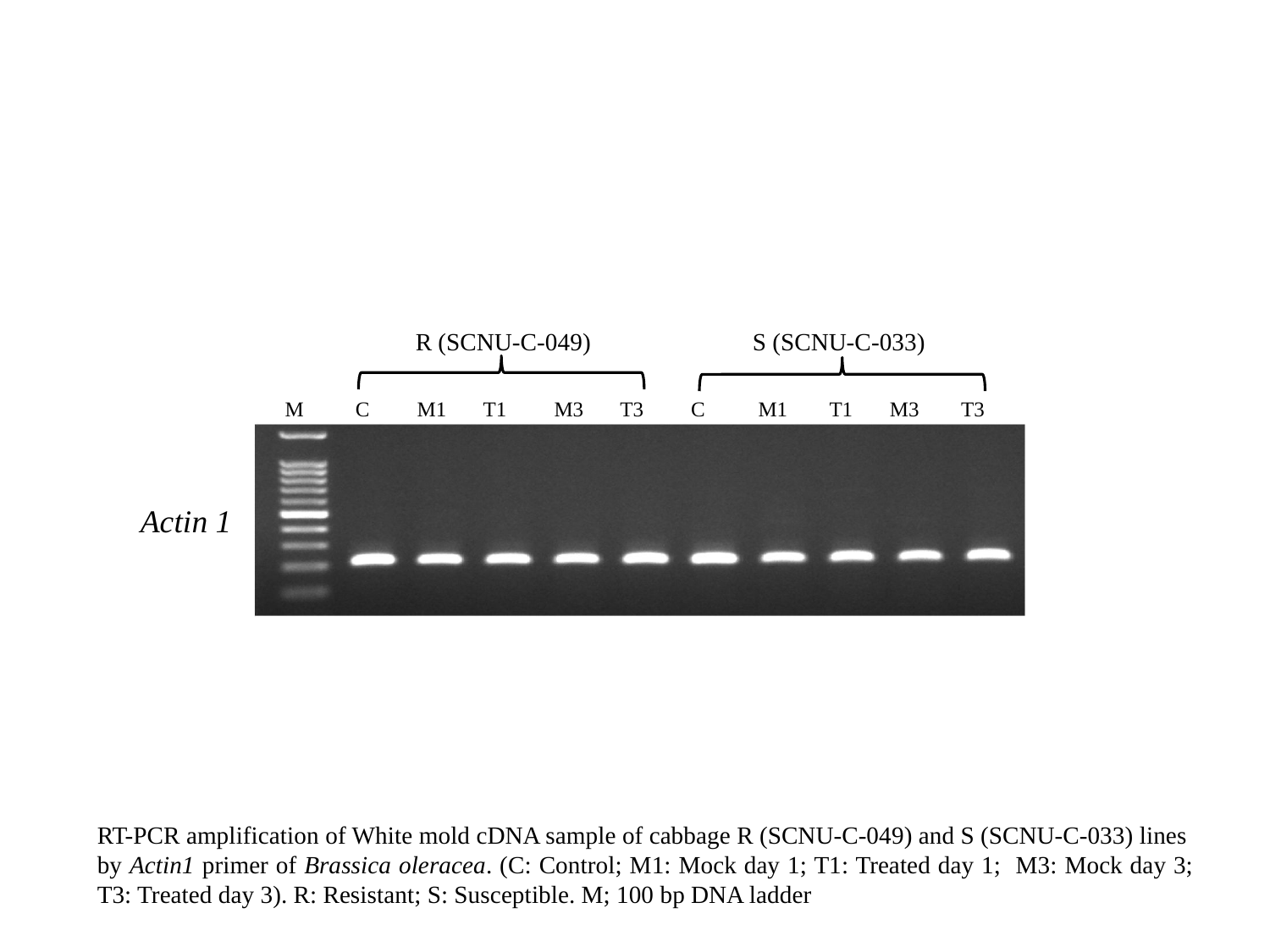

R (SCNU-C-049)
S (SCNU-C-033)
C M1 T1 M3 T3 C M1 T1 M3 T3
M
Actin 1
RT-PCR amplification of White mold cDNA sample of cabbage R (SCNU-C-049) and S (SCNU-C-033) lines by Actin1 primer of Brassica oleracea. (C: Control; M1: Mock day 1; T1: Treated day 1; M3: Mock day 3; T3: Treated day 3). R: Resistant; S: Susceptible. M; 100 bp DNA ladder
