# Supplementary material for: Glucosinolate Profiling and Expression Analysis of Glucosinolate Biosynthesis Genes Differentiate White Mold Resistant and Susceptible Cabbage Lines
Source: Int J Mol Sci. 2018 Dec 13;19(12):4037. doi: 10.3390/ijms19124037 (PMC6321582; doi:10.3390/ijms19124037)
Supplement: Supplementary file 1 [file ijms-19-04037-s001.zip › Supplementary tables.docx]

**Table S1.** Heat maps comparing white-mold-resistant (R; SCNU-C-049) and -susceptible (S; SCNU-C-033) lines shows fold changes in idividual glucosinolate component in *Sclerotinia sclerotiorum*–inoculated leaf samples compared to respective mock-treated samples. D1 (Day 1), T1/M1; D3 (Day 3), T3/M3; M1, mock day 1; T1, treated day 1; M3, mock day 3; T3, treated day 3.

| **Glucosinolate component** | **Treatments** | **SCNU-C-049** | **SCNU-C-033** |
| --- | --- | --- | --- |
| Glucoiberin (GIB) | D1 | 0.43 | 0.67 |
|  | D3 | 0.42 | 0.53 |
| Progoitrin (PRO) | D1 | 2.04 | 1.05 |
|  | D3 | 0.16 | 0.51 |
| Glucoraphanin (GRA) | D1 | 0.41 | 0.89 |
|  | D3 | 0.15 | 0.61 |
| Sinigrin (SIN) | D1 | 0.74 | 0.84 |
|  | D3 | 0.12 | 0.52 |
| Gluconapin (GNA) | D1 | 1.01 | 2.15 |
|  | D3 | 1.47 | 2.34 |
| Glucoiberverin (GIV) | D1 | 0.97 | 0.87 |
|  | D3 | 1.46 | 0.93 |
| Glucoerucin (GER) | D1 | 1.08 | 1.70 |
|  | D3 | 1.19 | 1.14 |
| Hydroxyglucobrassicin (HGBS) | D1 | 1.01 | 1.06 |
|  | D3 | 0.81 | 1.06 |
| Glucobrassicin (GBS) | D1 | 0.68 | 5.50 |
|  | D3 | 5.28 | 0.39 |
| Methoxyglucobrassicin (MGBS) | D1 | 1.16 | 1.96 |
|  | D3 | 1.17 | 1.26 |
| Neoglucobrassicin (NGBS) | D1 | 1.11 | 1.54 |
|  | D3 | 0.58 | 0.99 |

**Table S2.** Heat maps comparing white-mold-resistant (R; SCNU-C-049) and -susceptible (S; SCNU-C-033) lines shows fold changes in expression of transcription factor related genes in *S. sclerotiorum*-inoculated leaf samples compared to respective mock-treated samples. D1 (Day 1), T1/M1; D3 (Day 3), T3/M3; M1, mock day 1; T1, treated day 1; M3, mock day 3; T3, treated day 3.

| Genes | Treatment | SCNU-C-049 | SCNU-C-033 |
| --- | --- | --- | --- |
| *MYB28-Bol007795* | D1 | 0.22 | 2.9 |
|  | D3 | 0.08 | 0.52 |
| *MYB28-Bol036286* | D1 | 0.11 | 21.6 |
|  | D3 | 0.72 | 0.23 |
| *MYB28-Bol017019* | D1 | 10.21 | 2.0 |
|  | D3 | 0.52 | 6.10 |
| *MYB28-Bol036743* | D1 | 0.03 | 5.8 |
|  | D3 | 0.24 | 0.03 |
| *MYB29-Bol008849* | D1 | 0.19 | 51.0 |
|  | D3 | 1.02 | 0.36 |
| *MYB34-Bol017062* | D1 | 3.08 | 0.7 |
|  | D3 | 0.23 | 0.26 |
| *MYB34-Bol007760* | D1 | 0.28 | 0.8 |
|  | D3 | 0.08 | 0.33 |
| *MYB34-Bol036262* | D1 | 1.86 | 3.9 |
|  | D3 | 0.64 | 0.43 |
| *MYB51-Bol013207* | D1 | 0.81 | 1.4 |
|  | D3 | 4.39 | 2.44 |
| *MYB51-Bol030761* | D1 | 12.04 | 0.3 |
|  | D3 | 5.80 | 3.49 |
| *MYB122-Bol026204* | D1 | 1.96 | 0.7 |
|  | D3 | 189.11 | 17.66 |

**Table S3.** Heat maps comparing white-mold-resistant (R; SCNU-C-049) and -susceptible (S; SCNU-C-033) lines shows fold changes in expression of aliphatic glucosinolate biosynthesis genes in *S. sclerotiorum*-inoculated leaf samples compared to respective mock-treated samples. D1 (Day 1), T1/M1; D3 (Day 3), T3/M3; M1, mock day 1; T1, treated day 1; M3, mock day 3; T3, treated day 3.

| Genes | Treatment | SCNU-C-049 | SCNU-C-033 |
| --- | --- | --- | --- |
| *ST5b-Bol026202* | D1 | 1.35 | 0.9 |
|  | D3 | 5.67 | 1.24 |
| *ST5b-Bol026201* | D1 | 1.22 | 0.8 |
|  | D3 | 0.22 | 0.51 |
| *GSL-OH-Bol033373* | D1 | 0.05 | 65.3 |
|  | D3 | 1.29 | 0.10 |
| *ST5c-Bol030757* | D1 | 0.38 | 1.1 |
|  | D3 | 68.59 | 3.36 |
| *FMOGS-OX2-Bol010993* | D1 | 0.07 | 5.5 |
|  | D3 | 0.08 | 0.18 |
| *FMOGS-OX5-Bol029100* | D1 | 0.82 | 1.1 |
|  | D3 | 0.12 | 0.68 |
| *FMOGS-OX5-Bol031350* | D1 | 0.83 | 1.7 |
|  | D3 | 0.12 | 0.93 |
| *AOP2-Bo2g102190* | D1 | 11.37 | 0.2 |
|  | D3 | 1.98 | 0.24 |
| *AOP2-Bo3g052110* | D1 | 0.31 | 8.4 |
|  | D3 | 1.75 | 0.15 |
| *AOP2-Bo9g006240* | D1 | 0.50 | 11.4 |
|  | D3 | 0.22 | 0.16 |

**Table S4.** Heat maps comparing white-mold-resistant (R; SCNU-C-049) and -susceptible (S; SCNU-C-033) lines shows fold changes in expression of indole glucosinolate-biosynthesis related genes in *S. sclerotiorum*–inoculated leaf samples compared to respective mock-treated samples. D1 (Day 1), T1/M1; D3 (Day 3), T3/M3; M1, mock day 1; T1, treated day 1; M3, mock day 3; T3, treated day 3.

| Genes | Treatment | SCNU-C-049 | SCNU-C-033 |
| --- | --- | --- | --- |
| *ST5a-Bol039395* | D1 | 0.66 | 1.7 |
|  | D3 | 25.30 | 1.00 |
| *ST5a-Bol026200* | D1 | 1.80 | 0.5 |
|  | D3 | 82.91 | 0.73 |
| *CYP81F1-Bol028913* | D1 | 0.42 | 1.6 |
|  | D3 | 0.73 | 1.79 |
| *CYP81F1-Bol028914* | D1 | 3.61 | 2.4 |
|  | D3 | 1.49 | 19.57 |
| *CYP81F1-Bol017375* | D1 | 0.18 | 2.0 |
|  | D3 | 1.24 | 0.44 |
| *CYP81F1-Bol017376* | D1 | 0.06 | 2.5 |
|  | D3 | 7.96 | 0.54 |
| *CYP81F2-Bol012237* | D1 | 7.00 | 0.6 |
|  | D3 | 48.32 | 12.47 |
| *CYP81F2-Bol014239* | D1 | 6.21 | 0.7 |
|  | D3 | 870.86 | 1135.44 |
| *CYP81F2-Bol026044* | D1 | 2.81 | 1.4 |
|  | D3 | 54.55 | 40.88 |
| *CYP81F3-Bol032711* | D1 | 0.78 | 0.4 |
|  | D3 | 1.08 | 0.10 |
| *CYP81F3-Bol028919* | D1 | 1.30 | 1.4 |
|  | D3 | 0.26 | 0.47 |
| *CYP81F4-Bol032712* | D1 | 0.81 | 3.7 |
|  | D3 | 21.12 | 14.47 |
| *CYP81F4-Bol032714* | D1 | 0.65 | 2.5 |
|  | D3 | 10.22 | 12.30 |
| *CYP81F4-Bol028918* | D1 | 0.64 | 0.5 |
|  | D3 | 0.69 | 0.06 |
| *IGMT1-Bol007029* | D1 | 0.55 | 0.1 |
|  | D3 | 24.66 | 10.30 |
| *IGMT1-Bol020663* | D1 | 94.92 | 0.4 |
|  | D3 | 181.31 | 652.47 |
| *IGMT2-Bol007030* | D1 | 5.08 | 0.2 |
|  | D3 | 170.90 | 41.08 |

**Table S5.** Component loadings of cabbage white-mold-resistant (R; SCNU-C-049) and -susceptible (S; SCNU-C-033) lines, glucosinolate component and glucosinolate biosynthesis pathway gene responses as determined by the principal component analysis (PCA).

| Variable | PC1 | PC2 | PC3 | PC4 |
| --- | --- | --- | --- | --- |
| **Glucosinolate components** | | | | |
| Glucoiberin | -0.324 | -0.244 | 0.079 | -0.314 |
| Progoitrin | 0.274 | -0.405 | -0.099 | -0.308 |
| Glucoraphanin | 0.292 | -0.377 | -0.045 | -0.353 |
| Sinigrin | -0.289 | -0.395 | 0.108 | -0.246 |
| Gluconapin | -0.044 | 0.114 | -0.765 | -0.123 |
| Glucoiberverin | 0.057 | 0.354 | 0.242 | -0.693 |
| 4-Hydroxyglucobrassicin | 0.254 | -0.434 | -0.158 | 0.147 |
| Glucoerucin | 0.380 | 0.113 | -0.243 | -0.162 |
| Glucobrassicin | 0.311 | 0.264 | 0.157 | -0.182 |
| 4-Methoxyglucobrassicin | 0.388 | 0.173 | 0.026 | -0.066 |
| Neoglucobrassicin | 0.202 | -0.196 | 0.436 | 0.171 |
| % Variation explained | 43.50 | 24.20 | 12.40 | 8.20 |
| **Cabbage lines** | **Mean PC scores (±SD)** | | | |
| R-line SCNU-C-049 | 1.904±1.212 a | 0.08±2.46 | 0.233±0.788 | 0.101±1.318 |
| S-line SCNU-C-033 | -1.904±1.097 b | -0.076±0.677 | -0.233±1.611 | -0.101±0.679 |
| P-value | 0.001 | 0.898 | 0.578 | 0.768 |

**Table S6.** Modifications of infection procedure compared to Zhao and Meng (2003) and Yu et al. (2010).

| **Zhao and Meng 2003** | **Yu et al. 2010** | **Our study** |
| --- | --- | --- |
| Detached leaf was infected at 9 to 12 leaf stages. | Detached leaves were infected. | Inoculated on live leaves at 9^th^ leaf stage |
| Two pieces of mycelial agar plug (5 mm in diameter) were used for infection | A mycelial agar plug (8 mm in diameter) was used for infection | One piece of mycelial plug (7 mm in diameter) was used for infection |
| Stems were inoculated one month before the sampling using a tooth-pick | Stems were inoculated two weeks after final flowering | Stems were inoculated at 9^th^ leaf with alpin |
| Inoculation was done at a height of 35 cm above the ground | Inoculation was done at a height of 50 cm above the ground | Inoculation was done at a height of 20 cm above the ground |
| Infected lesion was measured at 5 days post inoculation (DPI) | Infected lesion was measured at 7 DPI | The lesion was measured from 0 to 5 DPI |
|  | The stem tooth-pick method was used for assessing the resistance in adult plants. | Cabbage heads were inoculated one month after head formation and then observed the head lesions at 10 DPI (with the head right side up) and at 15 DPI (with the head upside down). |

**Table S7.** Primer sequences and efficiency [[35](#_ENREF_35),[57](#_ENREF_57)] for the 38 glucosinolate biosynthesis related genes used in the relative expression analysis through qPCR in white-mold-resistant (R; SCNU-C-049) and -susceptible (S; SCNU-C-033) lines.

| **Gene  Name** | **Accession  Number** | **cDNA  Size (bp)** | **Forward Primer Sequence** | **Reverse Primer Sequence** | **Product  Size (bp)** | **Primer efficiency values (%)** |
| --- | --- | --- | --- | --- | --- | --- |
|  | Transcription factor-related genes (11 genes) | | | | | |
| ***MYB28*** | Bol007795 | 558 | CCACACCAGTTCAGAGAGGT | GGGAAATGGATCGAAGTCAGC | 221 | 98 |
|  | Bol036286 | 615 | GAAGGTAGCTTGAATGCTAATAC | ATTCATGTAGTGCTCCTCATTC | 249 | 98 |
|  | Bol017019 | 426 | GTTGCGGCTAAGGTCACTTCT | CAGAAGTAGCGTTGATCTCATGC | 223 | 99 |
|  | Bol036743 | 426 | CTTGGGCGCTGCTACATTAC | ATCGTTCTCCTCGTTGTGGT | 241 | 97 |
| ***MYB29*** | Bol008849 | 513 | CGCCCAAGACTTCTGAGTT | TGATATTGCCCATGGAAGCTG | 234 | 95 |
| ***MYB34*** | Bol007760 | 843 | TG‍AAGGAGGATGGCGTACTC | CAGTTCGTCCCGCCAAATTA | 203 | 86 |
|  | Bol017062 | 951 | AAGGTGGATGGCGTACTCTC | TGTGAGTGGTTGGATCGACA | 279 | 98 |
|  | Bol036262 | 294 | ATGTCGACATCTTCGGGTTT | CCAAGAATCAAGAAACTCCA | 222 | 96 |
| ***MYB51*** | Bol013207 | 1002 | GGTGAAGAATAGCAACAAGA | TTCGGAGTTAACGGTGACAC | 184 | 99 |
|  | Bol030761 | 990 | CGTGGATTACCGGGAAGAAC | TCTTCATTCTTGACCTTCTC | 227 | 97 |
| ***MYB122*** | Bol026204 | 981 | GACCATTCCGAGACATTGCC | GCATCGTGGATCATGTGGAG | 284 | 94 |
|  | Aliphatic biosynthesis-related genes (10 genes) | | | | | |
| ***ST5b*** | Bol026201 | 1035 | CCGAGCCGTCAGAATTCAAG | GCTATGGCGAAAGTGAGAGC | 247 | 92 |
|  | Bol026202 | 1035 | AAGCCTTGACTTTCGCCATC | ACTTCACAACTGAGTCCGGT | 204 | 100 |
| ***ST5c*** | Bol030757 | 1014 | CCACGCCCAAAACTTCTTCA | TGAGTGGAGAAGAGCGTGTT | 246 | 99 |
| ***FMOGS-OX2*** | Bol010993 | 1386 | GAGAAGGTATCCGAGCCACA | GTCCACTGCAAACAACGACT | 200 | 98 |
| ***FMOGS-OX5*** | Bol029100 | 1347 | CTTGCTCCAACGCTTTCCTT | CCTCAGCTCTCCAGTGTTCA | 280 | 92 |
|  | Bol031350 | 1380 | ATGGCACCCTCTTGCAGTCC | AGTCGTAGACGCTAGAGTGG | 226 | 99 |
| ***AOP2*** | Bo2g102190 | 1104 | GGAACGTGTCTCCAAAACCC | TAGCACCATCACCAGCATCA | 354 | 92 |
|  | Bo3g052110 | 948 | ATGGGTTCACACAGTACTCC | GGCCTCAACAGGTAGCTGGA | 216 | 99 |
|  | Bo9g006240 | 1032 | ATGGGTGCAGACACTCCTCA | TAGCCTCAACTGGTAACTCG | 214 | 98 |
| ***GSL-OH*** | Bol033373 | 243 | GATTGTGCAAAAGGCTTGT | AGAGCATTAGGATTAGGAGGA | 188 | 96 |
|  | Indolic biosynthesis-related genes (17 genes) | | | | | |
| ***ST5a*** | Bol026200 | 1017 | GTCCGGTTGCAAGATGGTTT | CCTCTCCGGGTTCTCTTTGT | 214 | 100 |
|  | Bol039395 | 1014 | TGCCGTTTGTGAAGAGGTTG | CCCAATCTCCAACCTTCCCT | 210 | 99 |
| ***CYP81F4*** | Bol032712 | 1506 | CGGTGGAGGAGAAGGAGAAA | CTGACACATGGCTCGTAACG | 226 | 98 |
|  | Bol032714 | 960 | ACCCTGGTGAATACTTGCCA | GAAACACACTGAAGCAGAAC | 239 | 98 |
|  | Bol028918 | 1503 | GTTTGCGGCATCAGAGACAT | GAATAGTCCACGCGTTCACC | 299 | 97 |
| ***CYP81F1*** | Bol017375 | 369 | AAGCAGAGCGGTTCAAGAAG | GCGTGACCATTGTGTTACCA | 204 | 95 |
|  | Bol017376 | 246 | CCGTCTCCTTCAACGGTTCT | CGACGTATTTACCGGTGAGC | 170 | 98 |
|  | Bol028913 | 1500 | GAGACCTCCGCAGTAACCTT | GTCCTCCGTCGGTCTTCTAG | 222 | 92 |
|  | Bol028914 | 1497 | CTTTCCAACTGACGGCCAAA | CGTTAGGTCCGAGAAAAGCG | 257 | 99 |
| ***CYP81F2*** | Bol012237 | 933 | GCAGCCGTGACACTAGAATG | TCCGCCAATCTTGAGGTCTT | 231 | 97 |
|  | Bol014239 | 1482 | TTGTACCGCGTTCTCCTTCT | GACACCATCCTCTGACCCAA | 238 | 95 |
|  | Bol026044 | 1482 | TCGGCAATCTCCACCTCGTG | GTCGCTCTGACCGGTGAAGC | 158 | 98 |
| ***CYP81F3*** | Bol028919 | 1500 | CGAGAAGAAAGTGAAAGCTG | TAAGGCCTTTGATAGTGACG | 164 | 98 |
|  | Bol032711 | 1491 | GTGAAAGCTGTTGGAGAAGC | GTTCCGGCGATCATCATGCC | 179 | 96 |
| ***IGMT1*** | Bol007029 | 1119 | GTGTTCCTCTCACCTTCCGA | GTGTTGAGGAAGACGCTGTC | 260 | 95 |
|  | Bol020663 | 342 | AGATGCCATGATCTTGAAACGT | CCAGCAATGATAAGCCTGACA | 298 | 90 |
| ***IGMT2*** | Bol007030 | 1125 | AGCCTTTCCCATGGTTCTCA | TCTCTCGCCCTTTCCAAACT | 223 | 92 |

**Table S8.** ANOVA for expression of glucosinolate biosynthesis genes in white-mold-resistant (R; SCNU-C-049) and -susceptible (S; SCNU-C-033) lines. Interactions; genotype-treatment- timepoint combinations.

| **Relative expression of genes** | **Source** | **DF** | **SS** | **MS** | **F-Value** | **P-Value** |
| --- | --- | --- | --- | --- | --- | --- |
| *MYB28-Bol007795* | Interactions | 9 | 64.3478 | 7.14976 | 334.67 | <0.01 |
|  | Error | 20 | 0.4273 | 0.02136 |  |  |
|  | Total | 29 | 64.7751 |  |  |  |
| *MYB28-Bol036286* | Interactions | 9 | 2405.91 | 267.323 | 733.77 | <0.01 |
|  | Error | 20 | 7.29 | 0.364 |  |  |
|  | Total | 29 | 2413.19 |  |  |  |
| *MYB28-Bol017019* | Interactions | 9 | 53.1999 | 5.9111 | 652.83 | <0.01 |
|  | Error | 20 | 0.1811 | 0.00905 |  |  |
|  | Total | 29 | 53.381 |  |  |  |
| *MYB28-Bol036743* | Interactions | 9 | 81.0824 | 9.00915 | 893.01 | <0.01 |
|  | Error | 20 | 0.2018 | 0.01009 |  |  |
|  | Total | 29 | 81.2842 |  |  |  |
| *MYB29-Bol008849* | Interactions | 9 | 1273.93 | 141.548 | 6164.79 | <0.01 |
|  | Error | 20 | 0.46 | 0.023 |  |  |
|  | Total | 29 | 1274.39 |  |  |  |
| *MYB34-Bol017062* | Interactions | 9 | 13.0037 | 1.44485 | 282.15 | <0.01 |
|  | Error | 20 | 0.1024 | 0.00512 |  |  |
|  | Total | 29 | 13.1061 |  |  |  |
| *MYB34-Bol007760* | Interactions | 9 | 4.86182 | 0.540202 | 108.06 | <0.01 |
|  | Error | 20 | 0.09998 | 0.004999 |  |  |
|  | Total | 29 | 4.96181 |  |  |  |
| *MYB34-Bol036262* | Interactions | 9 | 2.36637 | 0.26293 | 186.09 | <0.01 |
|  | Error | 20 | 0.02826 | 0.001413 |  |  |
|  | Total | 29 | 2.39463 |  |  |  |
| *MYB51-Bol013207* | Interactions | 9 | 2.92729 | 0.325254 | 445.52 | <0.01 |
|  | Error | 20 | 0.0146 | 0.00073 |  |  |
|  | Total | 29 | 2.94189 |  |  |  |
| *MYB51-Bol030761* | Interactions | 9 | 4.8302 | 0.536689 | 77.93 | <0.01 |
|  | Error | 20 | 0.1377 | 0.006887 |  |  |
|  | Total | 29 | 4.9679 |  |  |  |
| *MYB22-Bol026204* | Interactions | 9 | 31520.4 | 3502.27 | 36243.59 | <0.01 |
|  | Error | 20 | 1.9 | 0.1 |  |  |
|  | Total | 29 | 31522.4 |  |  |  |
| *ST5a-Bol039395* | Interactions | 9 | 284.134 | 31.5704 | 245.05 | <0.01 |
|  | Error | 20 | 2.577 | 0.1288 |  |  |
|  | Total | 29 | 286.71 |  |  |  |
| *ST5a-Bol026200* | Interactions | 9 | 343.326 | 38.1473 | 729.43 | <0.01 |
|  | Error | 20 | 1.046 | 0.0523 |  |  |
|  | Total | 29 | 344.372 |  |  |  |
| *ST5b-Bol026202* | Interactions | 9 | 265.565 | 29.5073 | 206.41 | <0.01 |
|  | Error | 20 | 2.859 | 0.143 |  |  |
|  | Total | 29 | 268.424 |  |  |  |
| *ST5b-Bol026201* | Interactions | 9 | 3825.35 | 425.039 | 415.91 | <0.01 |
|  | Error | 20 | 20.44 | 1.022 |  |  |
|  | Total | 29 | 3845.79 |  |  |  |
| *ST5c-Bol030757* | Interactions | 9 | 148.074 | 16.4526 | 674.29 | <0.01 |
|  | Error | 20 | 0.488 | 0.0244 |  |  |
|  | Total | 29 | 148.562 |  |  |  |
| *FMOGS-OX2-Bol010993* | Interactions | 9 | 42.2762 | 4.69736 | 1311.72 | <0.01 |
|  | Error | 20 | 0.0716 | 0.00358 |  |  |
|  | Total | 29 | 42.3478 |  |  |  |
| *FMOGS-OX5-Bol029100* | Interactions | 9 | 5.4467 | 0.60519 | 22.07 | <0.01 |
|  | Error | 20 | 0.5485 | 0.02743 |  |  |
|  | Total | 29 | 5.9952 |  |  |  |
| *FMOGS-OX5-Bol031350* | Interactions | 9 | 17.794 | 1.97711 | 60.97 | <0.01 |
|  | Error | 20 | 0.6485 | 0.03243 |  |  |
|  | Total | 29 | 18.4425 |  |  |  |
| *GSL-OH-Bol033373* | Interactions | 9 | 115.191 | 12.7989 | 83.05 | <0.01 |
|  | Error | 20 | 3.082 | 0.1541 |  |  |
|  | Total | 29 | 118.273 |  |  |  |
| *CYP81F1-Bol028913* | Interactions | 9 | 3.2553 | 0.3617 | 36.93 | <0.01 |
|  | Error | 20 | 0.1959 | 0.009794 |  |  |
|  | Total | 29 | 3.4512 |  |  |  |
| *CYP81F1-Bol028914* | Interactions | 9 | 37386.9 | 4154.1 | 1730.52 | <0.01 |
|  | Error | 20 | 48 | 2.4 |  |  |
|  | Total | 29 | 37434.9 |  |  |  |
| *CYP81F1-Bol017375* | Interactions | 9 | 11.214 | 1.24601 | 23.19 | <0.01 |
|  | Error | 20 | 1.075 | 0.05373 |  |  |
|  | Total | 29 | 12.289 |  |  |  |
| *CYP81F1-Bol017376* | Interactions | 9 | 4.87871 | 0.542079 | 1263.21 | <0.01 |
|  | Error | 20 | 0.00858 | 0.000429 |  |  |
|  | Total | 29 | 4.8873 |  |  |  |
| *CYP81F2-Bol012237* | Interactions | 9 | 1865.66 | 207.295 | 2169.68 | <0.01 |
|  | Error | 20 | 1.91 | 0.096 |  |  |
|  | Total | 29 | 1867.57 |  |  |  |
| *CYP81F2-Bol014239* | Interactions | 9 | 499708 | 55523.1 | 17480.11 | <0.01 |
|  | Error | 20 | 64 | 3.2 |  |  |
|  | Total | 29 | 499772 |  |  |  |
| *CYP81F2-Bol026044* | Interactions | 9 | 2416.33 | 268.481 | 2504.42 | <0.01 |
|  | Error | 20 | 2.14 | 0.107 |  |  |
|  | Total | 29 | 2418.48 |  |  |  |
| *CYP81F3-Bol032711* | Interactions | 9 | 141.676 | 15.7418 | 76.83 | <0.01 |
|  | Error | 20 | 4.098 | 0.2049 |  |  |
|  | Total | 29 | 145.773 |  |  |  |
| *CYP81F3-Bol028919* | Interactions | 9 | 2.9994 | 0.333267 | 74.27 | <0.01 |
|  | Error | 20 | 0.08975 | 0.004487 |  |  |
|  | Total | 29 | 3.08915 |  |  |  |
| *CYP81F4-Bol032712* | Interactions | 9 | 10097.7 | 1121.97 | 4449.06 | <0.01 |
|  | Error | 20 | 5 | 0.25 |  |  |
|  | Total | 29 | 10102.8 |  |  |  |
| *CYP81F4-Bol032714* | Interactions | 9 | 23504 | 2611.55 | 3810.37 | <0.01 |
|  | Error | 20 | 13.7 | 0.69 |  |  |
|  | Total | 29 | 23517.7 |  |  |  |
| *CYP81F4-Bol028918* | Interactions | 9 | 40.7063 | 4.52292 | 341.91 | <0.01 |
|  | Error | 20 | 0.2646 | 0.01323 |  |  |
|  | Total | 29 | 40.9709 |  |  |  |
| *IGMT1-Bol007029* | Interactions | 9 | 17.7219 | 1.9691 | 221.24 | <0.01 |
|  | Error | 20 | 0.178 | 0.0089 |  |  |
|  | Total | 29 | 17.8999 |  |  |  |
| *IGMT1-Bol020663* | Interactions | 9 | 2.83362 | 0.314846 | 1967.95 | <0.01 |
|  | Error | 20 | 0.0032 | 0.00016 |  |  |
|  | Total | 29 | 2.83682 |  |  |  |
| *IGMT2-Bol007030* | Interactions | 9 | 26.272 | 2.91911 | 10540.68 | <0.01 |
|  | Error | 20 | 0.0055 | 0.00028 |  |  |
|  | Total | 29 | 26.2775 |  |  |  |
| *AOP2-Bo2g102190* | Interactions | 9 | 2.44147 | 0.271275 | 453.6 | <0.01 |
|  | Error | 20 | 0.01196 | 0.000598 |  |  |
|  | Total | 29 | 2.45343 |  |  |  |
| *AOP2-Bo3g052110* | Interactions | 9 | 1178.04 | 130.893 | 1426.69 | <0.01 |
|  | Error | 20 | 1.83 | 0.092 |  |  |
|  | Total | 29 | 1179.87 |  |  |  |
| *AOP2-Bo9g006240* | Interactions | 9 | 160671 | 17852.3 | 8009.92 | <0.01 |
|  | Error | 20 | 45 | 2.2 |  |  |
|  | Total | 29 | 160716 |  |  |  |

**Table S9.** ANOVA for glucosinolate component in white-mold-resistant (R; SCNU-C-049) and – susceptible (S; SCNU-C-033) lines. Interactions; genotype-treatment- timepoint combinations.

| **Glucosinolate component** | **Source** | **DF** | **SS** | **MS** | **F-Value** | **P-Value** |
| --- | --- | --- | --- | --- | --- | --- |
| Glucoiberin (GIB) | Interactions | 9 | 4106.4 | 456.267 | 87.55 | <0.01 |
|  | Error | 20 | 104.2 | 5.211 |  |  |
|  | Total | 29 | 4210.6 |  |  |  |
| Progoitrin (PRO) | Interactions | 9 | 63.8086 | 7.08984 | 335.69 | <0.01 |
|  | Error | 20 | 0.4224 | 0.02112 |  |  |
|  | Total | 29 | 64.231 |  |  |  |
| Glucoraphanin (GRA) | Interactions | 9 | 144.341 | 16.0379 | 652.07 | <0.01 |
|  | Error | 20 | 0.492 | 0.0246 |  |  |
|  | Total | 29 | 144.833 |  |  |  |
| Sinigrin (SIN) | Interactions | 9 | 6685.92 | 742.88 | 232.86 | <0.01 |
|  | Error | 20 | 63.8 | 3.19 |  |  |
|  | Total | 29 | 6749.72 |  |  |  |
| Gluconapin (GNA) | Interactions | 9 | 33.814 | 3.75707 | 47.06 | <0.01 |
|  | Error | 20 | 1.597 | 0.07984 |  |  |
|  | Total | 29 | 35.41 |  |  |  |
| Glucoiberverin (GIV) | Interactions | 9 | 4.52 | 0.50222 | 11.02 | <0.01 |
|  | Error | 20 | 0.9114 | 0.04557 |  |  |
|  | Total | 29 | 5.4314 |  |  |  |
| Glucoerucin (GER) | Interactions | 9 | 1.825 | 0.20274 | 2.76 | 0.028 |
|  | Error | 20 | 1.471 | 0.07353 |  |  |
|  | Total | 29 | 3.295 |  |  |  |
| Hydroxyglucobrassicin (HGBS) | Interactions | 9 | 0.02585 | 0.002873 | 1.53 | 0.204 |
|  | Error | 20 | 0.03755 | 0.001877 |  |  |
|  | Total | 29 | 0.0634 |  |  |  |
| Glucobrassicin (GBS) | Interactions | 9 | 1685.18 | 187.242 | 395.06 | <0.01 |
|  | Error | 20 | 9.48 | 0.474 |  |  |
|  | Total | 29 | 1694.66 |  |  |  |
| Methoxyglucobrassicin (MGBS) | Interactions | 9 | 24.001 | 2.66674 | 35.43 | <0.01 |
|  | Error | 20 | 1.506 | 0.07528 |  |  |
|  | Total | 29 | 25.506 |  |  |  |
| Neoglucobrassicin (NGBS) | Interactions | 9 | 270.86 | 30.095 | 12.07 | <0.01 |
|  | Error | 20 | 49.89 | 2.494 |  |  |
|  | Total | 29 | 320.74 |  |  |  |
